# An integrated approach to biomarker discovery reveals gene signatures highly predictive of cancer progression

**DOI:** 10.1038/s41598-020-78126-3

**Published:** 2020-12-04

**Authors:** Kevin L. Sheng, Lin Kang, Kevin J. Pridham, Logan E. Dunkenberger, Zhi Sheng, Robin T. Varghese

**Affiliations:** 1 Edward Via College of Osteopathic Medicine, 2265 Kraft Drive, Blacksburg, VA 24060 USA; 2 Fralin Biomedical Research Institute at VTC, 2 Riverside Circle, Roanoke, VA 24016 USA; 3 Department of Internal Medicine, Virginia Tech Carilion School of Medicine, Roanoke, VA 24016 USA; 4 Faculty of Health Science, Virginia Tech, Blacksburg, VA 24061 USA

**Keywords:** Cancer, Biomarkers

## Abstract

Current cancer biomarkers present variability in their predictive power and demonstrate limited clinical efficacy, possibly due to the lack of functional relevance of biomarker genes to cancer progression. To address this challenge, a biomarker discovery pipeline was developed to integrate gene expression profiles from The Cancer Genome Atlas and essential survival gene datasets from The Cancer Dependency Map, the latter of which catalogs genes driving cancer progression. By applying this pipeline to lung adenocarcinoma, lung squamous cell carcinoma, and glioblastoma, genes highly associated with cancer progression were identified and designated as progression gene signatures (PGSs). Analysis of area under the receiver operating characteristics curve revealed that PGSs predicted patient survival more accurately than previously identified cancer biomarkers. Moreover, PGSs stratified patients with high risk for progressive disease indicated by worse prognostic outcomes, increased frequency of cancer progression, and poor responses to chemotherapy. The robust performance of these PGSs were recapitulated in four independent microarray datasets from Gene Expression Omnibus and were further verified in six freshly dissected tumors from glioblastoma patients. Our results demonstrate the power of an integrated approach to cancer biomarker discovery and the possibility of implementing PGSs into clinical biomarker tests.

## Introduction

Cancer is the second leading cause of death in the United States with over 600,000 fatalities in 2019 despite recent advances in management and treatment^1^. Among cancer types, malignancies in the central nervous system and the lung remain the leading causes of cancer-associated death and present dismal 5-year survival rates of 4.7% and 16%, respectively^2–6^. Current methods for predicting cancer progression include patient performance scoring and the American Joint Commission on Cancer Tumor-Node-Metastasis (TNM) staging system, which are primarily evaluated through physical exams and imaging^7–10^. However, the static formulation of both methods on clinicopathologic factors fails to account for the genetic heterogeneity of cancer^9,10^, limiting their predictive value. The inaccurate classification of patient risk for progressive disease selects cancer patients for ineffective treatment regimens, thereby contributing to high incidences of tumor progression that are directly associated with poor prognoses in central nervous system and lung cancers^4,11–13^. Thus, it is imperative to develop novel biomarkers for cancer progression to guide effective therapeutic intervention.

Molecular biomarkers predictive of progressive disease have been used for improving cancer management. Mutation and expression of epidermal growth factor receptor (*EGFR*) and Kirsten rat sarcoma viral oncogene homolog (*KRAS*) in lung cancer and promoter methylation of O^6^-methylguanine DNA methyltransferase (*MGMT*) in glioblastoma (GBM) have been identified to be associated with progressive disease and treatment response^3,6,13–15^. However, these current biomarkers do not fully represent the complex mechanisms of cancer progression in lung cancer and GBM. Recent studies have identified multiple *EGFR*- and *KRAS*-independent mechanisms for lung cancer progression including upregulation of mesenchymal-epithelial transition factor (*MET*)^16^, and progressive disease eventually develops in GBM patients regardless of *MGMT* promoter methylation status^17^. Accordingly, multiple reports have found the prognostic and predictive significance of *EGFR* and *KRAS* to vary between studies^18–21^, and a separate meta-analysis by Binabaj et al. demonstrated an insignificant association between promoter methylation of *MGMT* and progression-free survival in GBM^22^. Additionally, the development of targeted therapies such as gefitinib for *EGFR*, salirasib for *KRAS*, and O^6^-benzylguanine for *MGMT* has failed to confer significant therapeutic benefits for lung cancer and GBM patients^13,15,20^, likely due to their inconsistent prognostic and predictive value^23^. The inaccurate prediction of cancer progression and failure of targeted therapies for current biomarkers is attributable to the distinct molecular heterogeneity characterizing cancer subtypes^24–26^, which cannot be fully captured using a single biomarker^26^.

To overcome this challenge, recent advances in high-throughput mRNA profiling techniques such as cDNA microarrays and RNA-sequencing (RNA-seq) have spurred the identification of prospective gene expression signatures associated with progressive disease^27–32^. For instance, Larsen et al. developed a 54-gene signature predictive of tumor recurrence in lung adenocarcinoma from genome-wide mRNA expression profiling^27^, and Chen et al. identified a seven-microRNA signature to detect recurrent disease in GBM^29^. In addition, the emergence of machine learning applications to RNA-seq data analysis has further augmented the discovery of cancer progression signatures. For example, Rueda et al. developed supervised machine learning models to identify multiple novel transcriptomic biomarkers predictive of prostate cancer progression^33,34^. However, most current prospective signatures have been found to be poorly reproducible^35–37^, likely due to the diverse clinical and technical factors across independent patient cohorts. Cross-cohort variability significantly influences current expression-based biomarker discovery approaches, rendering putative biomarkers identified from these methods sensitive to overfitting as gene expression patterns observed in one cohort are not always representative of the population^38,39^. Therefore, the development of more robust screening approaches for candidate biomarkers is needed.

Gene function and its relevance in the context of cancer progression may provide useful fundamental information for biomarker candidate screening to overcome cross-cohort variability. Particularly, functional information about gene relevance to cancer cell survival can reveal molecular implications for candidate biomarkers in cancer progression that cannot be identified from gene expression analyses. The emergence of loss-of-function screens using RNA interference (RNAi) has developed a powerful high-throughput tool for determining gene functions and activities relevant to cell survival in cancer^40^. Accordingly, we and others have previously implemented genome-wide RNAi screens for therapeutic target discovery in cancer^41–44^. Interestingly, a number of so-called “survival genes” identified via RNAi have also been identified as prognostic biomarkers in these studies. Varghese et al.^42^ or Goidts et al.^43^ employed RNAi screens to discover survival genes in GBM, which subsequently led to the identification of phosphoinositol 3-kinase catalytic subunit β as a predictive biomarker of GBM recurrence or 6-phosphofructo-2-kinase/fructose-2,6-biphosphatase 4 as a GBM prognostic biomarker. The same approach has also identified chromodomain helicase DNA binding protein 4 as a survival gene and prognostic biomarker for breast cancer in other studies^44,45^. However, there has not been a comprehensive integration of RNAi screens into traditional expression-based biomarker discovery approaches to identify genes associated with cancer cell survival as biomarkers of cancer progression. With the release of The Cancer Dependency Map (DepMap), which includes Project Achilles from Broad Institute, a large-scale effort aimed at completing RNAi screens in over 2000 cancer cell lines^46^, we are offered the opportunity to utilize survival gene screens as an important additional factor in discovering novel candidate biomarkers of cancer progression.

Based on what was described above, current approaches to biomarker discovery have several limitations in capturing the molecular heterogeneity of cancer, particularly the neglect of genes essential for cancer cell survival as an important factor in predicting tumor progression. To address this issue, we report a novel biomarker discovery pipeline which integrates genome-wide RNAi screens from DepMap with comprehensive RNA-seq and clinical data from The Cancer Genome Atlas (TCGA) to identify survival gene-based progression gene signatures (PGSs). We selected one common cancer subtype (lung adenocarcinoma, LUAD), one less common cancer subtype (lung squamous cell carcinoma, LUSC), and one rare cancer type (GBM) to evaluate the feasibility of the pipeline regardless of cancer occurrence rate. Applying this pipeline revealed LUAD-PGS, LUSC-PGS, and GBM-PGS, respectively. Further investigation in multiple patient cohorts and freshly dissected tumor tissues verified the significance of these gene signatures as biomarkers of cancer progression. Given that these genes are not only essential for cancer survival but also correlated with cancer prognosis, our integrated approach to cancer biomarker discovery demonstrates important impacts on cancer diagnosis and therapeutic intervention.

## Methods

### Retrieval and analysis of patient gene expression and clinical data

The TCGA database contains publicly-accessible, RSEM-processed RNA sequencing (RNA-seq) data for 500+ quality-controlled primary tumor samples in LUAD and LUSC and genome-wide microarray profiling for 528 quality-controlled primary GBM samples. Gene expression and corresponding clinical data for 517 LUAD, 501 LUSC, and 528 GBM patients were retrieved from cBioPortal^47,48^ and used as the training set. To compile the NSCLC validation cohort, datasets from the NCBI Gene Expression Omnibus repository were screened for microarray chip type (Affymetrix U133 Plus 2.0, GPL570), availability of LUAD and LUSC samples, and availability of overall survival (OS) or disease-free survival (DFS) status and time-to-event data. Raw data from four selected microarray datasets (GSE3141^49^, GSE8894^50^, GSE19188^51^, and GSE30219^52^) were downloaded and pre-processed using robust multiarray averaging for normalization, then compiled to form validation cohorts that include 246 LUAD or 207 LUSC patients, respectively. In GBM, a random sampling technique stratified on age and gender was used to separate the TCGA cohort into a 396-patient training and 132-patient validation cohort due to the limited availability of external datasets. Microarray profiling and clinical data for 200 GBM patients from Rembrandt^53^ were retrieved to use as an independent validation cohort. Additionally, OS status and time-to-event data for six primary GBM samples obtained from patients who underwent surgical resection at Carilion Clinic were retrieved for experimental validation. These patients were de-identified and the IRB protocol was approved by Carilion Clinic IRB office. Available clinical characteristics for each cohort are summarized in Supplementary Table S1. Unstratified survival of all training and validation cohorts are shown in Supplementary Figure S1.

### Analysis of RNAi screen data from the Cancer Dependency Map database

The DepMap database contains data from the Project Achilles initiative by Broad Institute. This database contains publicly accessible, genome-wide RNAi screen results across 501 cancer cell lines^46^, including 18 NSCLC and 20 GBM cell lines. The screens include over 50,000 short hairpin RNAs (shRNAs) targeting the human genome and present results as log_2_ fold change of shRNA depletion. RNAi results from the Achilles 2.20.2 release were retrieved from DepMap and pre-processed to calculate the average log_2_ fold change across all shRNAs targeting each gene in each cell line.

### Isolation and culture of primary GBM cells

The use of human GBM patient specimens has been approved by the Institutional Review Board at Carilion Clinic and we confirm that informed consent was obtained from all participants and/or their legal guardians as required in the IRB. Freshly resected human GBM tumors (pathologically confirmed) were minced into small pieces. Single cells were prepared using Liberase (Roche Diagnostics) according to the manufacturer's instructions. Red blood cells were removed using Red Blood Cell Lysis Solution purchased from Miltenyi Biotec Inc. Isolated cells were cultured in DMEM (Life Technologies) supplemented with 15% FBS (Peak Serum, Inc.), streptomycin (100 μg/mL), and penicillin (100 IU/ml), (Life Technologies Corporation). Primary GBM cells were kept at no more than 10 passages.

### Identification of PGSs

Comprehensive RNA-seq or microarray data for over 500 patients in the TCGA training cohort were first used to identify the most ubiquitously expressed genes in two predominant NSCLC subtypes, LUAD and LUSC, and in GBM. A 99th-percentile cutoff was initially employed to ensure mRNA detection in other gene expression profiling platforms, resulting in the selection of 200 genes. This cutoff was further refined to 100 genes after downstream Bayesian Information Criterion (BIC) score optimization of the resulting gene signatures (Supplementary Table S2). Genes from this primary candidate pool were subsequently cross-referenced in 18 NSCLC or 20 GBM cell lines with available genome-wide RNAi screen data through Project Achilles. Since Project Achilles presents RNAi results as log_2_ fold changes indicative of shRNA loss, lower fold change values confer a stronger depletion of shRNAs and, thus, a larger reduction in cell viability following target gene knockdown. An average shRNA fold change cutoff of < 0 was implemented to select survival genes associated with cancer cell survival. One-tailed one-sample *t*-tests determined the significance of fold change < 0 for each shRNA, and Fisher’s combined probability test confirmed the false discovery rate (FDR)-adjusted significance of average shRNA fold change < 0. Genes not present in the Project Achilles database were excluded from further analyses. All survival genes were then entered into a backward stepwise variable regression model trained on a yes/no indicator of tumor progression incidence with a *p*-value threshold of 0.25 for PGS assembly.

### Derivation of PGS risk scores for patient risk stratification

Tumor progression risk scores were derived by a combination of statistical and machine-learning approaches. Principal component analysis (PCA) was first used to generate a set of principal components (PCs) linearizing z-score-normalized gene expression values across each PGS for each patient. The number of PCs generated was equal to the number of genes in each PGS. Each PC set was then screened using random forests of 1000 trees trained on a yes/no indicator of tumor progression incidence to select PCs highly correlated with progression incidence, implementing a percent contribution cutoff of > 0.05. Selected PCs were entered into a second PCA, and the process was iterated until random forests retained all PCs. The end PC set was entered into a neural network with three tanH nodes boosted 100 times at a 0.1 learning rate with tenfold cross validation. The resulting formula output the predicted probability of tumor progression on a scale of 0 to 1, which were then transposed to a scale of − 50 to 50 for ease of interpretation. A cutoff at 0 stratified patients as high-risk progression (> 0) or low-risk progression (< 0).

### Assessment of PGS risk score accuracy

The accuracy of patient risk stratification determined by each PGS was evaluated using various statistical methods. The frequency of tumor progression events within each risk group were calculated within confusion matrices, and significance testing of correlations were evaluated with Fisher’s Exact Tests. The area under the receiver operating characteristic (ROC) curve (AUC) values were interpreted as the fraction of accurately predicted cases. Pair-wise comparison of ROC curves fit using PGS-derived risk scores or current progression biomarkers determined significance of accuracy improvement. Kaplan–Meier survival analyses and Cox proportional hazards models determined association of patient risk groups with DFS time.

### Correlation analysis of PGS-stratified risk and treatment response

Clinical data on adjuvant chemotherapy (ACT) or TMZ administration for the TCGA training cohorts were retrieved from the NCI GDC data portal, and the Buffa hypoxia scores^54^ for each patient were retrieved from TCGA PanCancer Atlas through cBioPortal. Differences in patient benefit from treatment across risk groups were assessed using one-tailed two-sample *t*-tests on unequal variances and Fisher’s Exact Tests. Two-tailed two-sample *t*-tests on unequal variances assessed the correlation of PGS risk stratification with tumor hypoxia in NSCLC.

### Validation of PGS and risk algorithm

The validation of both NSCLC PGSs was accomplished via a retrospectively-compiled cohort of four independent microarray datasets, while GBM-PGS was validated in both an internal TCGA validation cohort and the external Rembrandt cohort. Gene expression data from each study were z-score normalized prior to risk algorithm application. NSCLC clinical data were processed as follows for cross-study compatibility: (1) Relapsed patients were categorized as “progressed” and non-relapsed patients “disease-free” in GS8894 and GSE30219; (2) Deceased patients were categorized as “progressed” and living patients as “disease-free” in GSE3141 and GSE19188, where relapse incidence data were unavailable. Accuracy of risk classification and characterization of risk groups were assessed using Fisher’s Exact Tests and Kaplan–Meier survival curves as described previously.

### Quantitative reverse transcription polymerase chain reaction (qRT-PCR)

Passage numbers for the six primary GBM cells are shown in Supplementary Table S3. Total RNA was isolated from frozen primary GBM cells using TriZol (Invitrogen), and cDNA was synthesized using reverse transcriptase (New England Biolabs). Primers (Sigma) were retrieved from literature search or PrimerBank and verified in Primer-BLAST (Supplementary Table S4). mRNA expression levels of GBM-PGS in six patient samples were measured by qRT-PCR using a StepOnePlus™ Real-Time PCR system. Glyceraldehyde-3-phosphate dehydrogenase (*GAPDH*) demonstrated the most stable expression compared to beta actin (*ACTB*) or beta 2 microglobulin (*B2M*) using RefFinder^55^ and was used as the control (Supplementary Table S5). ∆Ct values were calculated by subtracting Ct values of genes of interest from the Ct value of *GAPDH* and z-score-normalized within the six GBM primary cells. The GBM-PGS risk algorithm was applied to the z-score-normalized ∆Ct values of each gene to calculate risk scores for each sample using the PCs and neural network trained on the GBM training cohort. Patients were stratified as high- or low-risk progression as described previously.

### Software and programs

Data preprocessing were performed in Microsoft Excel and R statistical software^56^. All statistical analyses and machine learning were conducted in JMP Pro 14.3 and Python 3.8.1.

## Results

### A novel biomarker identification pipeline reveals PGSs in lung cancer and GBM

**Figure 1:**
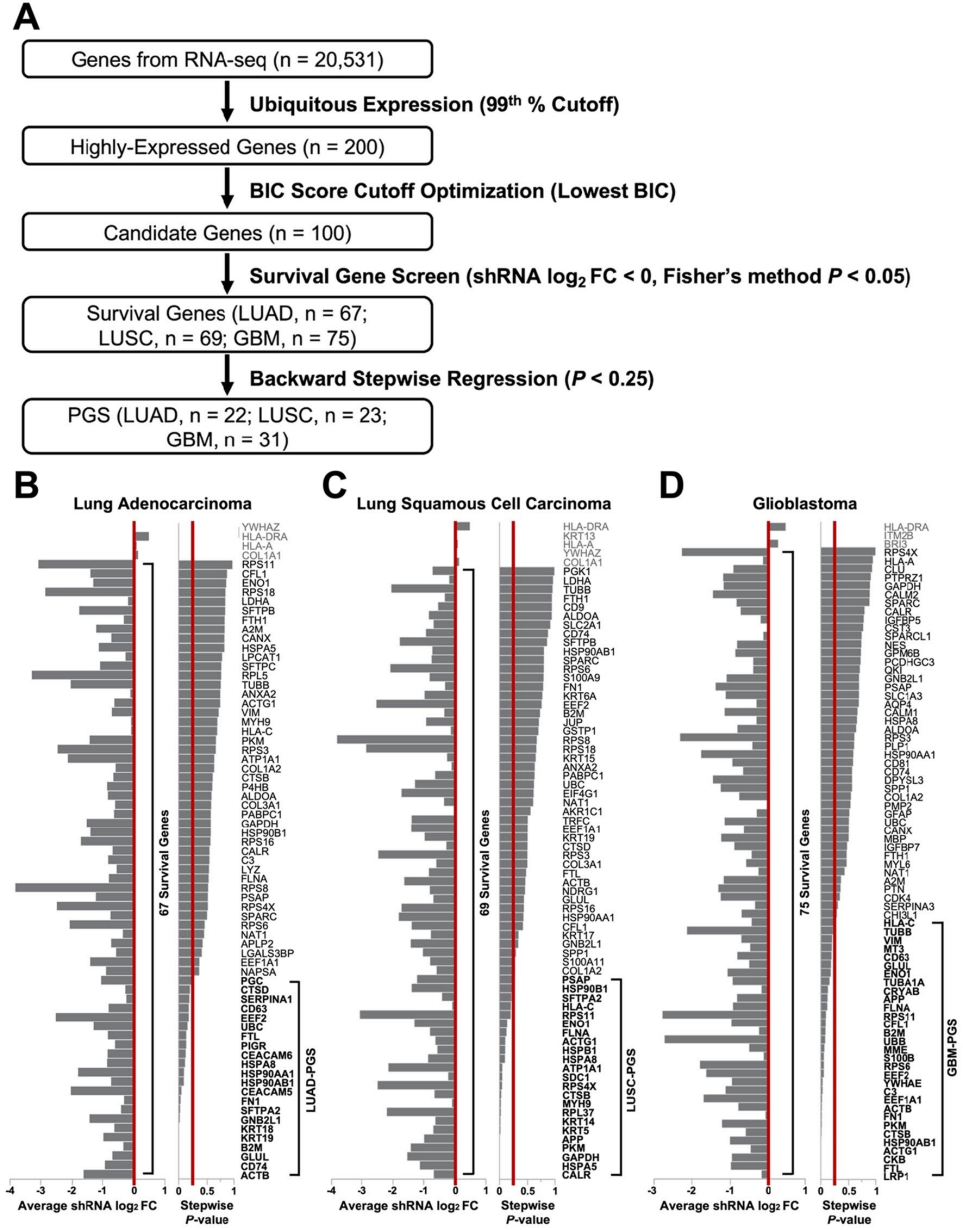
A novel biomarker discovery pipeline identifies new sets of PGS in lung cancer and GBM. (**A**) A schematic flowchart illustrating step-wise a biomarker discovery pipeline. log_2_ fold change values of shRNA depletion for the top 100 most ubiquitously expressed genes in LUAD (**B**), LUSC (**C**), and GBM (**D**) were calculated from Project Achilles and shown in the left panels. 29, 26, and 22 genes were undetected in Project Achilles for LUAD, LUSC, and GBM, respectively, and excluded from downstream analyses. A fold change cutoff of < 0 (red line, left panels) was used to select genes essential for cancer cell survival. One-sample one-tailed *t*-tests and Fisher’s method determined the significance of fold change < 0 (Supplementary Table S5). Survival genes were then entered into a backward stepwise variable regression model and selected to form PGSs using an arbitrary *p*-value threshold of 0.25 (red line, right panels) to select for interacting variables. Each survival gene and their corresponding stepwise *P*-values are shown in the right and middle panels, respectively.

To address challenges in identifying reliable cancer biomarkers, we developed a novel working pipeline (Fig. 1A) for the identification of cancer progression biomarkers. First, comprehensive RNA-seq or microarray data in TCGA were used to identify the most ubiquitously expressed genes in two predominant NSCLC subtypes, LUAD and LUSC, and in GBM. A 99^th^-percentile cutoff resulted in a candidate pool of 200 genes. This cutoff was further refined to 100 genes after using Bayesian Information Criterion (BIC) score optimization of the resulting gene signatures (Supplementary Table S2). These 100 genes were subsequently cross-referenced in 18 NSCLC or 20 GBM cell lines with available genome-wide RNAi screen data through DepMap. Since DepMap presents RNAi results as log_2_ fold changes indicative of shRNA loss, lower fold change values confer a stronger depletion of shRNAs and, thus, a larger reduction in cell viability following target gene knockdown. An shRNA fold change cutoff of < 0 was implemented to select survival genes associated with cancer cell survival. One-tailed one-sample *t*-tests and Fisher’s combined probability test confirmed the FDR-adjusted significance of shRNA fold change < 0 (Supplementary Table S6). Genes not present in the DepMap database were excluded from further analyses. All survival genes were then entered into a backward stepwise variable regression model trained on a yes/no indicator of tumor progression incidence with a *p*-value threshold of 0.25 for PGS assembly. This new pipeline allows us to test a novel hypothesis that genes essential for the survival of cancer cells are important candidate biomarkers for predicting disease progression.

By using the pipeline described in Fig. 1A and a cutoff of average shRNA log_2_ fold change < 0 (red lines), 67, 69, and 75 survival genes were identified in LUAD, LUSC and GBM, respectively (Fig. 1B–D, left panels). These highly expressed survival genes were then collectively assessed for their correlation with tumor progression incidence to assemble PGSs as biomarkers. Using backwards stepwise variable regression, *P*-values indicating the significance of candidate genes as predictor variables of tumor progression incidence in the model were calculated (Fig. 1B–D, right panel). By employing a *P*-value threshold of 0.25 (red lines), which allows us to select potential interacting variables that increase performance, a 22-gene LUAD-PGS, 23-gene LUSC-PGS, and 31-gene GBM-PGS were revealed (Fig. 1B–D, highlighted in bold, and Supplementary Table S7-S9). Interestingly, there was only a 2-gene overlap between PGSs identified in LUAD and LUSC. To further characterize these distinct signatures, we investigated the mutation frequency of PGS genes in the TCGA cohorts. Almost all genes in the NSCLC PGSs were mutated in at least one patient (Supplementary Table S10-S11). Kaplan–Meier survival analyses revealed that mutations in eukaryotic translation elongation factor 2 (*EEF2*) in LUAD-PGS or cathepsin B (*CTSB*) and heat shock protein 90 beta family member 1 (*HSP90B1*) in LUSC-PGS correlated with shorter disease-free survival (DFS) time (Supplementary Fig. S2A-C). These results demonstrate the difference of molecular profiles among NSCLC subtypes and a critical need for novel biomarkers to monitor disease progression in these subtypes. In GBM-PGS, signature genes were less frequently mutated compared to the NSCLC PGSs (Supplementary Table S12). Despite the low mutation frequency, mutations in amyloid beta precursor protein (*APP*) and membrane metalloendopeptidase (*MME*) significantly correlated with shorter DFS time (Supplementary Fig. S2D–E).

The above PGSs were selected from genes essential for cancer cell survival; hence, it is likely that they are closely associated with cancer-related signaling pathways that control cancer cell proliferation and survival. To determine the functional relevance among these PGSs and validate their roles in tumor growth and progression, we queried the Reactome program^57^ to assess the enrichment of PGSs in molecular pathways. As summarized in Table 1, PGSs were heavily enriched in various immune response pathways associated with cancer development and progression. Genes in LUAD-PGS were highly involved in neutrophil degranulation, a process known to be associated with tumor plasticity and cancer metastasis^58^. In contrast, signature genes in LUSC-PGS or GBM-PGS were associated with cytokine signaling, which is implicated in regulating cellular proliferation and survival^59^. We next queried STRING, a program that determines potential protein–protein interactions (PPI)^60^. The number of edges, which describes the interconnectivity among a specified gene set, were 59, 66, and 123 in PPI networks of LUAD-PGS (22 genes), LUSC-PGS (23 genes), and GBM-PGS (31 genes), respectively, demonstrating significant interconnectivity between signature genes (Table 1, *P* < 0.0001). Taken together, these results demonstrate the functional and physical connections among PGSs that are important for cancer growth and progression.

**Table 1:** PGSs are highly enriched in cancer-associated pathways and form significant protein–protein interaction networks. The three most relevant pathways from Reactome pathway analysis are shown in the left panel. Protein–protein interaction (PPI) networks were constructed using STRING and summarized in the right panel. The number of edges describes the level of interconnectivity of the networks and is expected to be equal to the number of genes in the network. *P*-values indicating whether the observed interactions were due to chance (PPI enrichment) were calculated by STRING.

	Reactome				STRING	
	Pathway	Number in pathway	Total genes in pathway	FDR *p*-value	Number of edges	PPI enrichment *p*-value
LUAD	Neutrophil degranulation	11	480	4.38e−6	59	7.36e−11
	Immune system	22	2803	4.38e−6		
	Interleukin-4 and Interleukin-13 signaling	6	211	0.001		
LUSC	Interferon signaling	16	392	2.70e−11	66	1.81e−13
	Cytokine signaling in immune system	23	1245	6.39e−10		
	Cell–cell communication	7	133	1.24e−05		
GBM	Interferon Signaling	19	392	1.83e−13	123	<1.00e−16
	Cytokine signaling in immune system	29	1245	2.67e−13		
	Gap junction trafficking	4	52	2.58e−03		

### PGS performance exceeds established biomarkers

**Figure 2:**
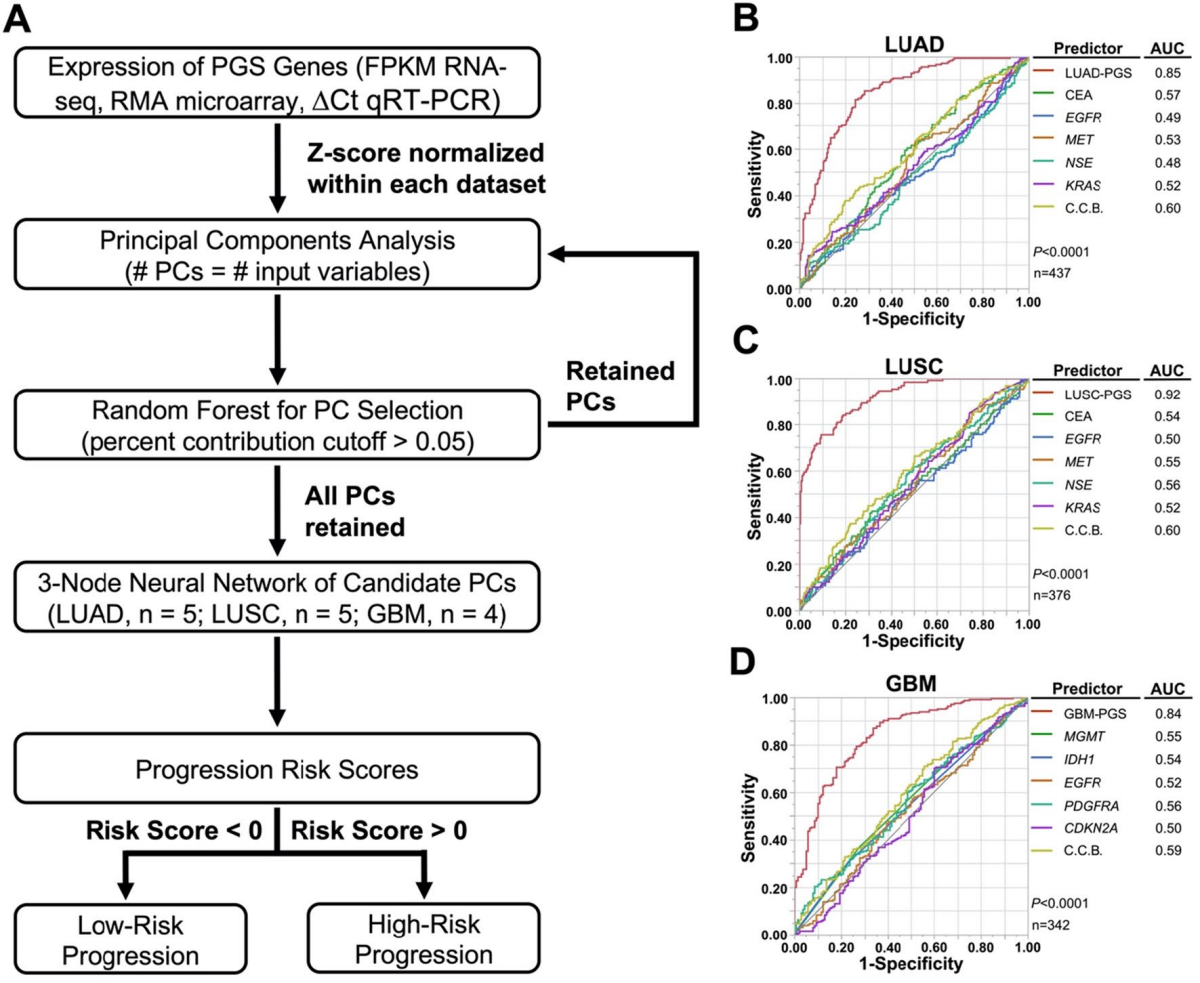
Prognostic significance between PGSs and established biomarkers. (**A**) A schematic flowchart illustrating a risk score algorithm to quantify patient risk for disease progression. ROC curves trained on PGS risk scores were used to calculate AUC values for LUAD-PGS (**B**), LUSC-PGS (**C**), and GBM-PGS (**D**) describing the overall accuracy of the model. Pair-wise comparisons were used to determine significance of PGS performance compared to current clinical biomarkers independently and in conjunction.

To determine the prognostic significance of PGSs, we developed a risk score algorithm linearizing patient expression levels of each PGS to quantify patient risk for disease progression. Risk scores for each patient in the TCGA training cohorts were calculated on a scale of -50 to + 50 representing lowest (− 50) to highest (+ 50) risk of progression. tenfold cross validation in the training cohorts resulted in AUC values of 0.85, 0.92, and 0.84 for LUAD-PGS (A), LUSC-PGS (B), and GBM-PGS (C), respectively (Fig. 2, red curves). We next determined the performance of established biomarkers such as the carcinoembryonic antigen (*CEA*) family, *EGFR*, tyrosine-protein kinase Met (*MET*), neuron-specific enolase (*NSE*), and *KRAS* for NSCLC^13,14,61,62^ and promoter methylation *of MGMT*, mutation of isocitrate dehydrogenase 1 (*IDH1*), *EGFR*, platelet-derived growth factor receptor alpha (*PDGFRA*), and cyclin-dependent kinase inhibitor 2A (*CDKN2A*) for GBM^15,63^. The AUC values of these established biomarkers ranged from 0.48 to 0.57 (Fig. 2, curves in different colors) and did not exceed 0.60 when assessed together (shown as combined current biomarkers; C.C.B.). These AUC values from established biomarkers were significantly lower than those of PGSs (*P* < 0.0001).

**Figure 3:**
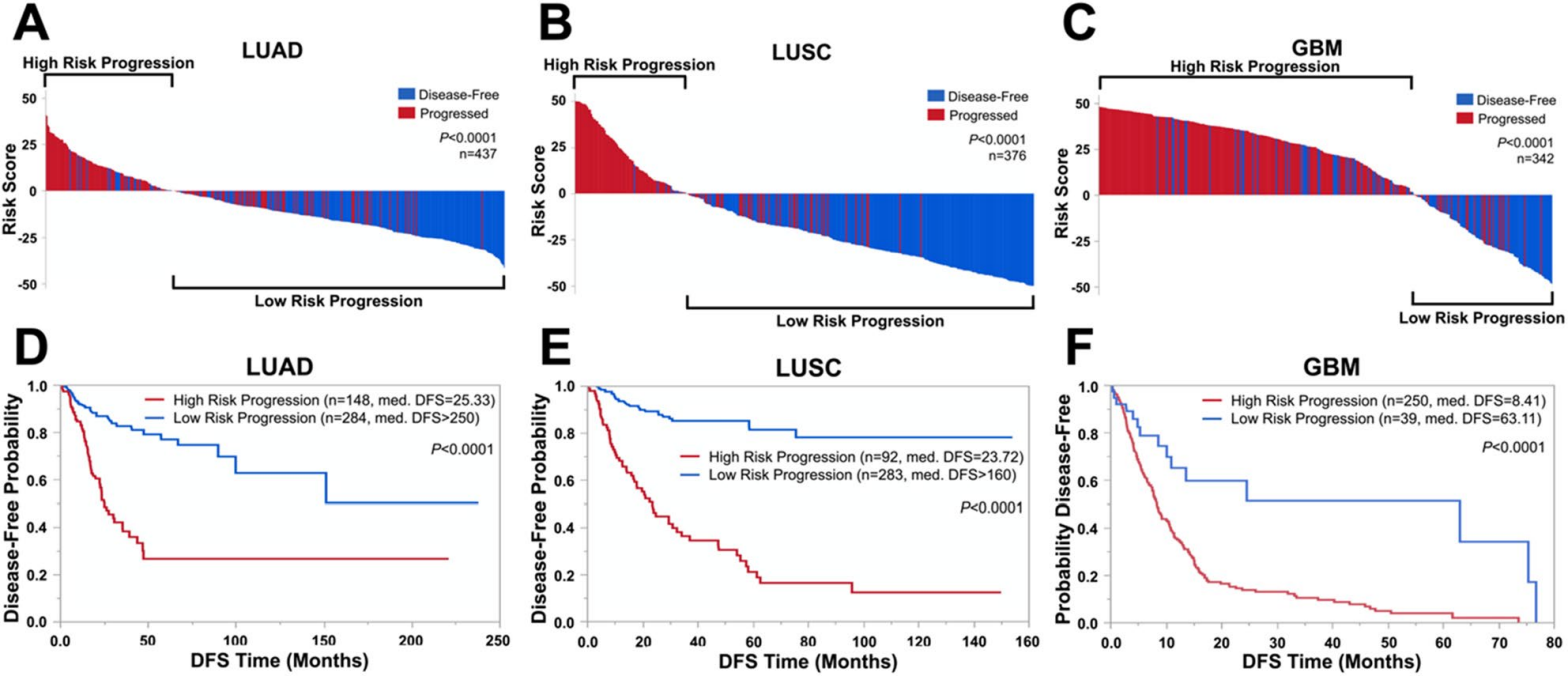
PGSs accurately stratify patients into risk groups correlating with tumor progression^60^. Patients were stratified as high-risk progression (risk score > 0) or low-risk progression (risk score < 0) and analyzed for correlations with tumor progression incidence. Fisher’s Exact Tests determined significance of correlation. (**D**–**F**) Kaplan–Meier survival curves of disease-free survival (DFS) time between high- and low-risk patients. Median DFS times for each risk group are shown in months. *P*-values were calculated using log-rank tests. C.C.B—combined current biomarkers, DFS—disease-free survival.

Next, we applied risk scores to stratify patients into high- or low-risk progression groups. A median risk score of 0 was used as the cutoff. As shown in Fig. 3, high-risk progression (risk score > 0) patients diagnosed with LUAD (A), LUSC (B) or GBM (C) exhibited significantly increased frequency of tumor progression (highlighted in red), whereas low-risk progression patients (risk score < 0) were mostly disease-free (blue). The *P*-value of this difference was less than 0.0001 in all cancers tested. Interestingly, patients harboring mutations in PGS genes that were prognostically significant were mostly classified as high-risk progression by all PGSs (Supplementary Fig. S3). Similar results were also observed in the classical (n = 107), mesenchymal (n = 112), and proneural (n = 170) GBM subtypes for high- and low-risk progression patients stratified by GBM-PGS (Supplementary Fig. S4A-C). Kaplan–Meier survival analyses revealed that LUAD (D), LUSC (E), or GBM (F) patients in the high-risk progression group presented much shorter life spans than patients in the low-risk progression group (Fig. 3D, P < 0.0001). The median DFS time in high-risk progression groups was 25.33 (Fig. 3D, LUAD), 23.72 (Fig. 3E, LUSC), or 8.41 (Fig. 3F, GBM) months. In stark contrast, median DFS times in low-risk progression groups were > 250 (LUAD), > 160 (LUSC), or 63.11 (GBM) months. We also analyzed DFS times of GBM-PGS risk groups in the three GBM subtypes to find that high-risk progression groups significantly correlated with worse patient prognosis in the mesenchymal (Supplementary Fig. S4E, *P* = 0.0104) and proneural (Supplementary Fig. S4F, *P* = 0.0008) subtypes but not the classical subtype (Supplementary Fig. S4D, *P* = 0.1337). The median DFS time in high-risk progression groups were 8.44 (classical), 7.1 (mesenchymal), and 8.21 (proneural) months compared to 15.9 (classical), 24.64 (mesenchymal), and 63.11 (proneural) months in low-risk progression patients.

To further determine the performance of PGSs in patient prognosis, we used Cox proportional hazards models. The hazard ratios (HRs), which indicate risk of death, of LUAD-PGS or LUSC-PGS were 5.07 or 6.91, respectively (Table 2, univariate). In contrast, HRs of Tumor-Node-Metastasis (TNM) stage, age, gender, or smoking history ranged from 0.57 to 2.34, which were significantly lower than the HRs of PGSs. Similarly, GBM-PGS was more significantly associated with tumor progression (HR = 3.02) than age or gender (HR = 1.02 or 1.04, respectively). To determine whether the prognostic potential of PGSs depends upon other factors, we performed Cox multivariate analysis. LUAD-PGS and LUSC-PGS presented prognostic significance independent of TNM stage, age, gender, or smoking history, and GBM-PGS was unrelated to age or gender in predicting patient prognosis (Table 2) because there was no significant difference between HRs of univariate (HR = 5.07, 6.91, or 3.02 for LUAD-PGS, LUSC-PGS, or GBM, respectively) and multivariate analyses (HR = 5.06, 6.57, or 2.90, respectively).

**Table 2:** PGSs are independent prognostic factors. Cox univariate and multivariate regression models were run using TNM stage, age, gender, and smoking history as additional clinicopathologic predictors for NSCLC and age and gender for GBM. Stage I-II patients were categorized as early-stage and stage III-IV patients were categorized as late-stage. The hazard ratios (HR), 95% confidence intervals (CI), and *P*-values are shown. TNM—Tumor-Node-Metastasis.

		Univariate			Multivariate		
		HR	95% CI	*P*	HR	95% CI	*P*
LUAD	LUAD-PGS	5.07	[3.42–7.58]	< 0.0001	5.06	[3.36–7.68]	< 0.0001
	TNM stage (Late vs. Early)	2.34	[1.50–3.56]	0.0003	2.36	[1.50–3.62]	0.0004
	Age	1.00	[0.98–1.02]	0.933	1.00	[0.98–1.02]	0.977
	Gender (Male vs. Female)	0.92	[0.62–1.35]	0.664	0.99	[0.66–1.46]	0.955
	Smoking History (Smoker vs. None)	0.99	[0.59–1.79]	0.982	0.99	[0.58–1.84]	0.992
LUSC	LUSC-PGS	6.91	[4.51–10.80]	< 0.0001	6.57	[4.23–10.41]	< 0.0001
	TNM stage (Late vs. Early)	2.23	[1.39–3.48]	0.001	1.69	[1.04–2.68]	0.034
	Age	1.02	[0.99–1.04]	0.07	1.02	[0.99–1.04]	0.07
	Gender (Male vs. Female)	1.27	[0.79–2.11]	0.335	0.99	[0.61–1.67]	0.962
	Smoking History (Smoker vs. None)	0.57	[0.21–2.31]	0.375	0.34	[0.12–1.40]	0.119
GBM	GBM-PGS	3.02	[1.78–5.63]	< 0.0001	2.90	[1.70–5.42]	< 0.0001
	Age	1.02	[1.01–1.03]	< 0.0001	1.02	[1.01–1.03]	0.0002
	Gender (Male vs. Female)	1.04	[0.79–1.39]	0.778	1.00	[0.75–1.34]	0.991

**Figure 4:**
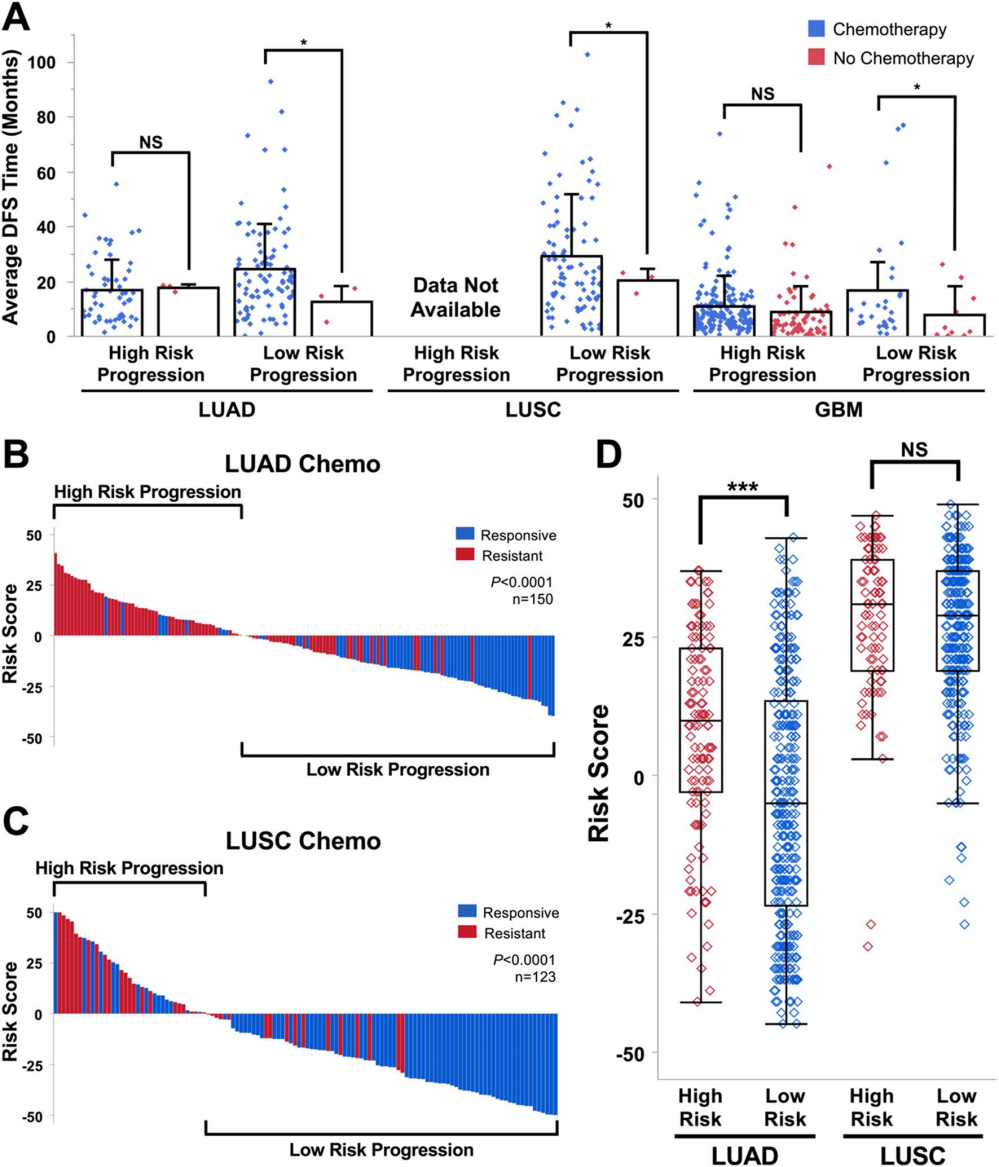
High-risk patients stratified by PGSs do not benefit from chemotherapy. (**A**) DFS of patients receiving ACT in NSCLC or TMZ in GBM. Average DFS times for each risk group are shown in months. *P*-values were calculated using student *t* tests. (**B**,**C**) Correlation of PGS risk stratification with patient response to ACT. Significance was determined using Fisher’s Exact Tests. (**D**) Buffa tumor hypoxia scores between PGS risk groups. Higher scores indicate hypoxia, while lower scores indicate normoxia. *P*-values were calculated using two-tailed *t*-tests on unequal variances. ***P < 0.0001, NS—not significant.

Treatment responses are often associated with tumor progression. ACT is the first-line therapy for NSCLC patients^6,13^, and TMZ is the only alkylating chemotherapeutic agent for GBM because of its efficient penetration through the blood–brain barrier^3,11^. However, ACT only presents a 4–15% survival advantage at 5 years post-treatment in early-stage NSCLC patients^64^, and around 50% of GBM patients develop resistance to TMZ and present poor prognosis^11^. To determine whether PGS-defined risk of poor prognosis correlates with treatment response, we analyzed the DFS times of high- and low-risk progression NSCLC patients treated with or without ACT or GBM patients treated with or without TMZ. The DFS times for high-risk progression patients treated with ACT or TMZ did not significantly differ compared to those treated without ACT or TMZ (Fig. 4A, P > 0.05). Of note, however, only three LUAD patients were treated without ACT in the high-risk progression group and included in these analyses. The average DFS times for high-risk progression patients treated with ACT or TMZ was 16.40 (LUAD) or 10.80 (GBM) months compared to 18.18 (LUAD) or 8.44 (GBM) months in patients treated without ACT or TMZ. Data were unavailable for LUSC due to a lack of high-risk progression patients treated without ACT. In contrast, DFS times for low-risk progression patients were significantly higher in patients treated with ACT or TMZ (Fig. 4A, P < 0.05). The average DFS times were 23.99 (LUAD), 28.86 (LUSC), and 16.52 (GBM) months in low-risk progression patients treated with ACT or TMZ compared to 12.28 (LUAD), 19.95 (LUSC), and 7.61 (GBM) months in patients treated without ACT or TMZ. While the sample sizes for LUAD and LUSC patients treated without ACT were small, these results suggest that patients with high risk of poor prognosis defined by PGSs may be resistant to chemotherapy. To further explore this observation, we retrieved ACT response information from the TCGA NSCLC cohorts. As expected, high-risk progression patients defined by PGSs were resistant to ACT, whereas low-risk progression patients were responsive to ACT (Fig. 4B–C, P < 0.0001). Next, we determined tumor hypoxia levels in high- or low-risk progression patients because hypoxia often induces ACT resistance^65^. Based upon hypoxia scores determined by the Buffa mRNA abundance signature, LUAD patients in the high-risk progression group (red) exhibited a greater incidence of hypoxia than patients in the low-risk progression group (blue) manifested by higher Buffa hypoxia scores (Fig. 4D, P < 0.0001). However, no difference in hypoxia score was detected in LUSC patients, possibly because the hypoxia index is already high in LUSC tumors^66^. Taken together, our results demonstrate that the PGSs identified herein are superior to established biomarkers in prognostic performance and that patients with high risk of poor prognosis, as defined by PGSs, are more likely to have shorter survival spans and develop progressive disease and therapeutic resistance.

### PGSs demonstrate robust performance in prognosis prediction in other patient cohorts and in freshly resected tumors of GBM patients

**Figure 5:**
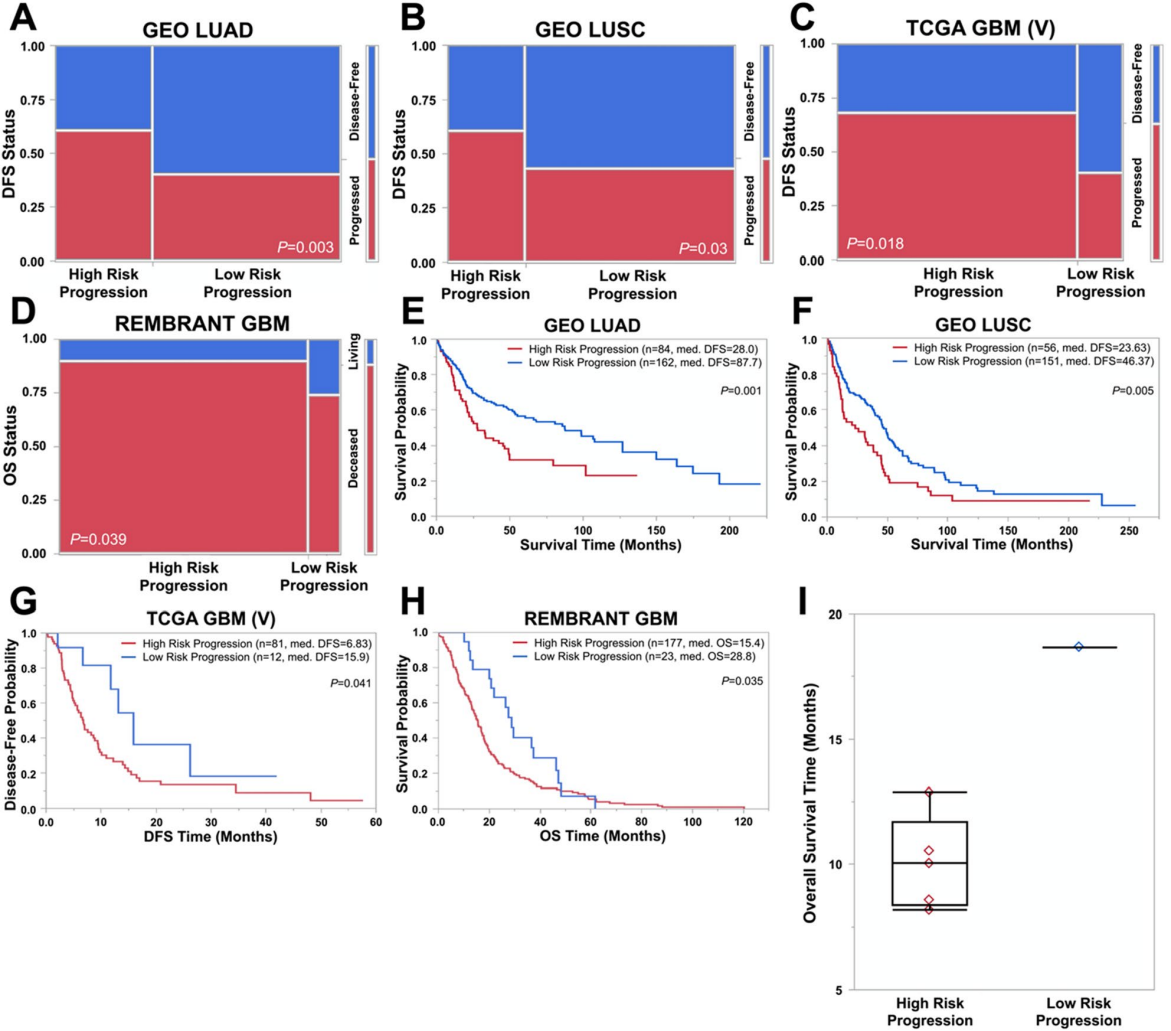
PGSs demonstrate robust performance in predicting prognosis in validation cohorts and GBM patients with freshly dissected tumors. (**A**,**B**) Patient risk stratification by LUAD-PGS and LUSC-PGS in a 246-patient and 207-patient validation cohort compiled from four independent microarray datasets from Gene Expression Omnibus (GEO). *P*-values were calculated via Fisher’s Exact Tests. (**C**,**D**) Patient risk stratification by GBM-PGS in a 126-patient TCGA validation cohort excluded from training and a 200-patient external validation cohort from Rembrandt. Overall survival (OS) status was used in Rembrandt due to a lack of progression data. *P*-values were calculated via Fisher’s Exact Tests. (**E**,**F**) Kaplan–Meier survival curves of survival time between high- and low-risk NSCLC patients. *P*-values were calculated using log-rank tests. (**G**,**H**) Kaplan–Meier survival curves of DFS time (**G**) or OS time (**H**) between high- and low-risk GBM patients. *P*-values were calculated using log-rank tests. (**I**) Primary cells were established from GBM tumor samples collected from Carilion Clinic. Expression of GBM-PGS genes were determined by RT-qPCR and analyzed using the GBM-PGS risk algorithm, stratifying five patients as high-risk (red) and one patient as low-risk (blue).

To validate the potential of PGSs identified herein as prognostic biomarkers, we retrieved four independent NSCLC microarray datasets from the Gene Expression Omnibus (GEO) database, a TCGA GBM validation cohort comprising 126 samples, and a 200-patient external GBM validation cohort from Rembrandt^53^. These patient cohorts are thereafter designated as validation cohorts. As expected, high-risk progression patients stratified by PGSs of LUAD (A), LUSC (B), or GBM (C) showed higher levels of tumor progression and lower levels of disease-free survival than low-risk progression patients (Fig. 5, *P* < 0.05). Consistently, data from the Rembrandt validation cohort showed that GBM patients in the high-risk progression group presented a greater chance of death than patients in the low risk progression group (Fig. 5D, P = 0.039). When GBM-PGS was analyzed in each GBM subtype, the high-risk progression group correlated with increased tumor progression in the mesenchymal and proneural subtypes (Supplementary Fig. S5B-C, *P* < 0.05) but not the classical subtype (Supplementary Fig. S5A, *P* = 0.3690). These results were further validated by Kaplan–Meier survival analyses. Median survival times in LUAD (Fig. 5E) or LUSC (Fig. 5F) patients with high risk of poor prognosis were 28 or 23.63 months, respectively. However, median survival times in patients with low risk of poor prognosis were much longer (87.70 months for LUAD and 46.37 months for LUSC). Similar results were obtained from the TCGA (Fig. 5G) and Rembrandt (Fig. 5H) GBM validation cohorts. The median DFS time in patients with high risk of poor prognosis was 6.83 and 15.4 months, which were significantly shorter than the median DFS time in patients with low risk of poor prognosis (15.2 or 28.8 months; *P* < 0.05). In the GBM subtypes, median DFS times for high-risk progression patients were 8.28 (classical), 6.7 (mesenchymal), and 6.685 (proneural) months compared to 21.04 (classical), > 45 (mesenchymal), and 12.435 (proneural) months for low-risk progression patients (Supplementary Fig. S5D-F). However, log-rank tests did not reveal statistical significance for these differences due to the low number of low-risk progression patients (n < 6) in all three GBM subtypes.

To prove the concept that PGSs are able to be used in clinical tests, we collaborated with the Fralin Biomedical Research Institute at Virginia Tech Carilion and Carilion Clinic and obtained six GBM primary lines derived from freshly dissected patient tumors. By employing quantitative RT-PCR to quantify mRNA levels of 31 genes in GBM-PGS and applying the risk algorithm defined in this study, five patients were stratified in the high-risk progression group and one patient in the low-risk progression group. As expected, patients in the group with high risk of poor prognosis presented an average OS time of 10.03 months, whereas the patient defined as low risk of poor prognosis survived for 18.68 months (Fig. 5I). While the sample size in this experiment was small, the capability of GBM-PGS in defining patients with high risk of poor prognosis was verified, thereby encouraging us to explore the potential of implementing PGSs into clinical tests. Hence, the results described above demonstrate the robustness of PGS performance in accurately predicting prognosis and highlight the potential of implementing PGSs into clinical tests.

## Discussion

In this report, we developed a novel biomarker discovery pipeline integrating genome-wide RNAi screens with global mRNA profiling data to identify survival gene-based PGSs in lung cancer and GBM. The importance of PGSs in predicting tumor progression, patient survival, and treatment response was further verified by multiple analyses in training cohorts and validation cohorts obtained from independent studies. Moreover, applying GBM-PGS in a small group of primary GBM samples mimicked a clinical test. Our innovative approach resulted in the identification of novel gene signatures that can be used as powerful prognostic markers for cancer diagnosis.

Tumor staging and performance scoring are two factors often used in the clinic for the prediction of patient outcomes and selection of patients for chemotherapies^7–10^. However, these two factors are not sufficient. Several recent studies have attempted to apply prospective gene signatures for better prediction of prognosis or therapeutic benefit with or without tumor staging and performance scoring; however, these studies lack a strong translational potential because they only employed gene expression-based approaches, neglecting the functional relevance of candidate genes to the disease. The novel biomarker pipeline described in this study identifies gene signatures based upon the importance of genes to cancer cell survival, which addresses the issue described above. While we only showed results in lung cancer and GBM, this pipeline could be a powerful tool in identifying biomarkers in other cancers.

The PGSs identified herein presented a robust performance in predicting patient outcomes that was superior to clinically-used biomarkers and molecular prognostic markers established previously, providing a strong support to our hypothesis. More importantly, we found that there was little overlap between the PGSs in this study and gene signatures in other studies^27,28,30–32^. For instance, we identified three heat shock protein (HSP) genes, HSP 90 alpha family class B member 1 (*HSP90AB1*), HSP family A member 8 (*HSPA8*), and HSP family A member 5 (*HSPA5*), as novel biomarkers in lung cancer and GBM. HSPs are diversely implicated in cell proliferation, invasion, and migration through their roles in controlling cell cycle progression and protecting cells against apoptosis under stress^67^. Certain HSP genes have been studied for association with patient prognosis and treatment response^67,68^; however, the HSP genes we identified have not been previously reported as lung cancer or GBM biomarkers. We also identified multiple cytoskeleton-associated genes, including keratin 18 (*KRT18*) in LUAD, keratin 14 (*KRT14*) in LUSC, and cofilin 1 (*CFL1*) in GBM as novel prognostic and predictive biomarkers. Past studies have highlighted the important role of cytoskeletal dynamics in mediating chemotherapy resistance and cancer metastasis^69^. Taken together, the functional relevance of PGSs to cancer cell survival, proliferation, and drug response further supports the feasibility of using essential survival genes as novel biomarkers that can accurately predict cancer progression.

The PGSs identified in this study contain some survival genes previously reported as prognostic markers. For example, carcinoembryonic antigen-related cell adhesion molecule 5/6 (*CEACAM5*/*CEACAM6*) in LUAD-PGS belongs to the well-known CEA protein family associated with carcinogenesis and progression in multiple cancers^61^. Fibronectin 1 (*FN1*) is a prognostic and predictive biomarker in head and neck squamous cell carcinoma^70,71^. Guanine nucleotide-binding protein subunit beta-2-like 1 (*GNB2L1*), also known as receptor for activated C kinase 1 (*RACK1*), serves as a prognostic biomarker in pancreatic and breast cancer^72,73^. Enolase 1 (*ENO1*) and cathepsin B (*CTSB*), found in both LUSC-PGS and GBM-PGS, are predictive biomarkers for hepatocellular carcinoma, gastric cancer, or oral squamous cell carcinoma^74–76^. The presence of established biomarkers within PGSs highlights the power and feasibility of our integrated approach to cancer biomarker discovery.

It is also noted that the construction of PGSs from genes implicated in cancer cell survival allows for the potential development of novel targeted therapies as companion therapeutics^41^. Accordingly, multiple signature genes in PGSs identified herein are appealing therapeutic targets worth further investigation. For instance, glutamate-ammonia ligase (*GLUL*) in LUAD-PGS and GBM-PGS encodes an enzyme catalyzing the synthesis of glutamine, an essential amino acid for DNA synthesis and repair^77^. Glutamine metabolism is often remodeled in cancer to increase cell proliferation^77,78^. Given the relatively low expression of *GLUL* in normal tissues^78^, the aberrant activity of *GLUL* in progressive cancer patients can be an appealing therapeutic target for LUAD and GBM. A GLUL inhibitor L-methionine-S,R-sulfoximine is commercially available^79^, and future studies should investigate the possibility of this inhibitor in treating LUAD or GBM. *CTSB* is a target candidate in LUSC-PGS and GBM-PGS, encoding a member of the cathepsin protein family which remodel the extracellular matrix to facilitate cancer invasion and metastasis^80^. A number of *CTSB* inhibitors have been developed^81^, but the efficacy of these drugs in lung cancer or GBM has not been explored. Some genes in LUSC-PGS or GBM-PGS were involved in interferon (IFN) signaling pathways. The roles of IFN signaling in tumors are controversial—IFN triggers anti-tumor immunity, but emerging evidence also suggest prolonged activation of IFN signaling leads to therapy resistance through increased *JAK*/*STAT* signaling^82^. As such, a number of *JAK*/*STAT* inhibitors including AZD1480 and LLL12 have demonstrated promising efficacy in treating NSCLC and GBM^83–85^. A recent study by Hu et al. also showed that the *JAK2* inhibitor ruxolitinib restored cisplatin sensitivity in NSCLC^86^. Taken together, our innovative biomarker discovery pipeline identifies PGSs that not only serve as accurate predictors of tumor progression and treatment response, but also help develop effective cancer therapies.

While our study unveils the feasibility of a novel approach integrating cancer cell survival and global mRNA profiling data for biomarker discovery, important questions remain to be addressed in order to facilitate the clinical implementation of PGSs in clinical diagnosis tests. Particularly, the retrospective nature of our study limited the sample size in analyzing the correlation between PGS risk groups and ACT response in NSCLC. While the observation that high-risk progression patients do not benefit from ACT was significant, this conclusion requires further validation. Future prospective studies are necessary to support the promising correlation between PGS risk groups and treatment response. As an important additional limitation, assaying GBM-PGS in six primary GBM cells stratified only one patient as low-risk progression and was not statistically conclusive. Our data proved the feasibility of implementing PGSs as clinical tests; however, large-scale clinical studies are required to statistically validate the capability of PGSs in defining patients with high risk of poor prognosis. Future studies will also aim to develop novel companion therapeutics for PGSs and additional biomarker discovery pipelines.

## Supplementary information

Supplementary Information
