# Supplementary material for: An integrated approach to biomarker discovery reveals gene signatures highly predictive of cancer progression
Source: Sci Rep. 2020 Dec 4;10:21246. doi: 10.1038/s41598-020-78126-3 (PMC7718261; doi:10.1038/s41598-020-78126-3)
Supplement: Supplementary file 1 — Supplementary Information [file 41598_2020_78126_MOESM1_ESM.docx]

**An Integrated Approach to Biomarker Discovery Reveals Gene Signatures Highly Predictive of Cancer Progression- SUPPLEMENTARY MATERIAL**

Kevin L. Sheng^1^, Lin Kang^1^, Kevin J. Pridham^2^, Logan E. Dunkenberger^2*^, Zhi Sheng^2,3,4^, Robin T. Varghese^1^

^1^Edward Via College of Osteopathic Medicine, 2265 Kraft Drive, Blacksburg, VA 24060, USA.

^2^Fralin Biomedical Research Institute at VTC, 2 Riverside Circle, Roanoke, VA 24016, USA.

^3^Department of Internal Medicine, Virginia Tech Carilion School of Medicine, Roanoke, VA 24016, USA.

^4^Faculty of Health Science, Virginia Tech, Blacksburg, VA 24061, USA.

*previous affiliation

**SUPPLEMENTARY TABLES**

**Table S1. Clinical characteristics of the training and validation cohorts.**

|  | **Training** | | | **Validation** | | | |  |
| --- | --- | --- | --- | --- | --- | --- | --- | --- |
| **Variables** | **TCGA LUAD** | **TCGA LUSC** | **TCGA GBM (T)** | **GEO LUAD** | **GEO LUSC** | **TCGA GBM (V)** | **REMBRT**  **GBM** | |
| No. of Patients | 517 | 504 | 389 | 246 | 207 | 126 | 200 | |
| Gender |  |  |  |  |  |  |  | |
| Male | 240 | 131 | 236 | 100 | 142 | 75 | 99 | |
| Female | 277 | 373 | 153 | 48 | 12 | 51 | 55 | |
| N/A | 0 | 0 | 0 | 98 | 53 | 0 | 46 | |
| Med. Age (Years) | 66 | 68 | 59 | 60.5 | 63.5 | 60 | 57 | |
| Race |  |  |  |  |  |  |  | |
| Caucasian | 393 | 351 | 341 |  |  | 112 | 139 | |
| African American | 53 | 31 | 22 |  |  | 8 | 2 | |
| Asian | 8 | 9 | 11 |  |  | 0 | 1 | |
| N/A | 63 | 113 | 15 | 246 | 207 | 6 | 58 | |
| AJCC TNM Stage |  |  |  |  |  |  |  | |
| Stage I | 279 | 245 |  | 81 | 48 |  |  | |
| Stage II | 124 | 163 |  | 3 | 5 |  |  | |
| Stage III | 85 | 85 |  | 1 | 2 |  |  | |
| Stage IV | 26 | 7 |  | 0 | 0 |  |  | |
| N/A | 3 | 4 |  | 161 | 152 |  |  | |
| Smoking History |  |  |  |  |  |  |  | |
| Non-Smoker | 75 | 18 |  |  |  |  |  | |
| Current Smoker | 122 | 134 |  |  |  |  |  | |
| Reformed Smoker | 307 | 335 |  |  |  |  |  | |
| N/A | 13 | 17 |  | 246 | 207 |  |  | |
| Subtype |  |  |  |  |  |  |  | |
| Classical |  |  | 107 |  |  | 36 |  | |
| Mesenchymal |  |  | 112 |  |  | 41 |  | |
| Proneural |  |  | 170 |  |  | 49 |  | |
| N/A |  |  | 0 |  |  | 0 | 200 | |

**Table S2. BIC score optimization to determine the cutoff for highly-expressed genes.** PGSs were identified using the working pipeline described in the Methods section. The cutoffs returning a PGS with the lowest BIC score, which corresponds to the lowest overfitting potential, are highlighted in grey.

|  | **Cutoff Value** | **Genes in PGS** | **BIC** |
| --- | --- | --- | --- |
| **LUAD** | 50 | 19 | 671.251 |
|  | **100** | **22** | **662.856** |
|  | 150 | 30 | 684.074 |
|  | 200 | 59 | 785.492 |
| **LUSC** | 50 | 20 | 567.156 |
|  | **100** | **23** | **559.984** |
|  | 150 | 34 | 580.935 |
|  | 200 | 53 | 635.626 |
| **GBM** | 50 | 28 | 778.526 |
|  | **100** | **31** | **767.708** |
|  | 150 | 48 | 819.793 |
|  | 200 | 48 | 802.173 |

**Table S3. Passage numbers for primary GBM cells.**

| **Primary GBM Cell** | **Passage Number** |
| --- | --- |
| VTC-001 | 3 |
| VTC-004 | 3 |
| VTC-010 | 5 |
| VTC-037 | 3 |
| VTC-058 | 5 |
| VTC-093 | 6 |

**Table S4. qRT-PCR primers used for detecting mRNA levels of PGS genes in GBM.**

| **Gene** | **Primers Sequence 5’-3’ (forward, reverse)** |
| --- | --- |
| *RPS11* | AGCAGCCGACCATCTTTC  ATAGCCTCCTTGGGTGTCTTG |
| *UBB* | AAGCCTAAACTGCCTCTC  GTTGCGTCACTTATCACC |
| *TUBB* | GCAGTCACCTTCATTGGCAAT  GCGGAACATGGCAGTGAACT |
| *RPS6* | CGCCAAGTATGTTGTAAGAAAGCCCT  GCTGCAGGACACGTGGAGTAACA |
| *EEF1A1* | CAGGACACAGAGACTTTATC  AGTGTGTAAGCCAGAAGGG |
| *EEF2* | AGAAGCTGTGGGGTGACAG  GATCAGCTGGCAGAAGGTG |
| *PKM* | GTGCGAGCCTCAAGTCACTCCACA  TATAAGAAGCCTCCACGCTGCCCA |
| *C3* | GCTGAAGCACCTCATTGTGA  CTGGGTGTACCCCTTCTTGA |
| *ENO1* | GAGCTCCGGGACAATGATAA  CTGTTCCATCCATCTCGATC |
| *HSP90AB1* | TTTGGGAACCATTGCCAAGTC  CACCAAACTGCCCAATCATGG |
| *FTL* | GCGTCTCCTGAAGATGCAAA  AGGAAGTGAGTCTCCAGGAAGT |
| *CFL1* | GAAGGAGGATCTGGTGTTTATCTTCT  CCTTGGAGCTGGCATAAATCAT |
| *YWHAE* | GGATACGCTGAGTGAAGAAAGC  TATTCTGCTCTTCACCGTCACC |
| *CKB* | GCTGCGACTTCAGAAGCGA  GGCATGAGGTCGTCGATGG |
| *TUBA1A* | GCAACAACCTCTCCTCTTCG  GAATCATCTCCTCCCCCAAT |
| *FLNA* | GCACTTACAGCTGCTCCTACG  CCAGCTCCCACATTCACC |
| *APP* | AACCAGTGACCATCCAGAAC  ACTTGTCAGGAACGAGAAGG |
| *CD63* | AGCAGATGGAGAATTACCC  CTCCCAATCTGTGTAGTTAG |
| *ACTB* | TTCCTGGGCATGGAGTC  CAGGTCTTTGCGGATGTC |
| *VIM* | AGCCGAAAACACCCTGCAAT  CGTTCAAGGTCAAGACGTGC |
| *CTSB* | GGCCCCCTGCATCTATCG  AGGTCTCCCGCTGTTCCACTG |
| *MME* | CATCGGCATGGTCATAGGACA  TGTTGAGTCCACCAGTCAACGA |
| *GLUL* | CACACAATCTTGGCATTTCC  ACTCAGGGGAGCAAAGGAAG |
| *MT3* | ATGGACCCTGAGACCTGCC  TTGCACACACAGTCCTTGGC |
| *ACTG1* | TGTTTCCTTCCATCGTCGGG  CATGTCGTCCCAGTTGGTGA |
| *HLA-C* | TCCTGGTTGTCCTAGCTGTC  CAGGCTTTACAAGTGATGAG |
| *B2M* | GATGAGTATGCCTGCCGTGT  TGCGGCATCTTCAAACCTCC |
| *CRYAB* | AGGTGTTGGGAGATGTGATTGA  GGATGAAGTAATGGTGAGAGGGT |
| *LRP1* | ACATATAGCCTCCATCCTAATC  TTCCAATCTCCACGTTCAT |
| *S100B* | AGACGGTCATGCAAGAAAGC  GCTACAACACGGCTGGAAAG |
| *FN1* | AGATCTACCTGTACACCTTGAATGACA  CATGATACCAGCAAGGAATTGG |

**Table S5. Stability of *GAPDH*, *ACTB*, and *B2M* expression in six primary GBM cell lines.** RefFinder assigns weights to reference gene stability rankings from four algorithms and calculates their geometric mean. Lower geometric means confer greater expression stability.

| **Gene** | **RefFinder** | **Comparative ∆Ct** | **BestKeeper** | **NormFinder** | **GeNorm** |
| --- | --- | --- | --- | --- | --- |
| ***GAPDH*** | **1.00** | **1.22** | **0.61** | **0.211** | **0.680** |
| *ACTB* | 1.68 | 1.27 | 0.87 | 0.647 | 0.680 |
| *B2M* | 3.00 | 1.80 | 1.32 | 1.738 | 1.429 |

**Table S6. Significance of shRNA log_2_ fold change <0 for survival genes.** One-tailed one-sample *t*-tests first analyzed the significance of fold change <0 for each shRNA targeting each candidate survival gene. False discovery rate-adjusted *P*-values (*Q*-values) were then calculated using Fisher’s combined probability test to determine the combined significance of all shRNA log_2_ fold change <0. Genes with average shRNA fold change >0 are shown in red.

| **LUAD** | | **LUSC** | | **GBM** | |
| --- | --- | --- | --- | --- | --- |
| **Gene** | ***Q*-value** | **Gene** | ***Q*-value** | **Gene** | ***Q*-value** |
| *RPS8* | 4.247E-43 | *RPS8* | 5.382E-43 | *RPS11* | 1.561E-61 |
| *RPL5* | 2.955E-55 | *RPS11* | 1.068E-55 | *UBB* | 5.165E-43 |
| *RPS11* | 1.054E-55 | *RPS18* | 1.442E-51 | *RPS3* | 1.870E-51 |
| *RPS18* | 9.482E-52 | *EEF2* | 3.447E-38 | *RPS4X* | 1.433E-38 |
| *EEF2* | 3.401E-38 | *RPS4X* | 2.489E-34 | *TUBB* | 4.876E-36 |
| *RPS4X* | 2.455E-34 | *RPS3* | 1.167E-43 | *RPS6* | 1.774E-39 |
| *RPS3* | 8.635E-44 | *RPL37* | 1.820E-38 | *HSP90AA1* | 7.630E-22 |
| *ATP1A1* | 1.896E-34 | *ATP1A1* | 1.922E-34 | *EEF1A1* | 4.590E-31 |
| *RPS6* | 6.508E-41 | *RPS6* | 7.917E-41 | *EEF2* | 2.763E-26 |
| *TUBB* | 1.127E-28 | *TUBB* | 1.142E-28 | *CALM2* | 3.081E-25 |
| *CEACAM5* | 5.806E-26 | *HSP90AA1* | 2.686E-26 | *DPYSL3* | 3.397E-25 |
| *HSP90AA1* | 2.650E-26 | *SFTPB* | 3.156E-26 | *PSAP* | 1.151E-17 |
| *SFTPB* | 3.113E-26 | *EIF4G1* | 4.571E-21 | *PTN* | 2.892E-16 |
| *RPS16* | 4.335E-27 | *RPS16* | 4.395E-27 | *CDK4* | 1.249E-26 |
| *ACTB* | 5.152E-17 | *ACTB* | 5.432E-17 | *SPP1* | 6.570E-19 |
| *GAPDH* | 1.312E-24 | *GAPDH* | 1.425E-24 | *MBP* | 5.758E-17 |
| *GNB2L1* | 9.111E-23 | *GNB2L1* | 9.852E-23 | *PKM* | 1.152E-17 |
| *PKM* | 1.012E-17 | *PKM* | 1.070E-17 | *PTPRZ1* | 5.770E-27 |
| *EEF1A1* | 1.451E-18 | *EEF1A1* | 1.471E-18 | *GAPDH* | 2.041E-19 |
| *CFL1* | 1.868E-22 | *TFRC* | 7.954E-16 | *A2M* | 1.674E-18 |
| *HSP90B1* | 2.593E-17 | *CFL1* | 2.012E-22 | *UBC* | 5.444E-19 |
| *ENO1* | 3.071E-18 | *HSP90B1* | 2.738E-17 | *CALM1* | 9.975E-13 |
| *UBC* | 7.652E-16 | *ENO1* | 3.113E-18 | *C3* | 5.265E-22 |
| *PSAP* | 7.165E-20 | *UBC* | 7.757E-16 | *SLC1A3* | 7.348E-21 |
| *A2M* | 4.804E-15 | *PSAP* | 7.263E-20 | *GNB2L1* | 8.158E-17 |
| *HSPA5* | 1.989E-04 | *HSPA5* | 1.989E-04 | *ENO1* | 6.686E-17 |
| *SFTPC* | 4.079E-12 | *SPP1* | 1.688E-13 | *HSP90AB1* | 4.738E-19 |
| *PGC* | 4.227E-19 | *APP* | 4.062E-07 | *FTL* | 5.026E-21 |
| *KRT19* | 3.572E-14 | *KRT6A* | 3.805E-19 | *CFL1* | 6.756E-19 |
| *CD74* | 4.827E-15 | *KRT19* | 4.205E-14 | *YWHAE* | 4.947E-15 |
| *NAPSA* | 9.726E-15 | *CD74* | 5.231E-15 | *CKB* | 1.484E-08 |
| *HSPA8* | 1.839E-13 | *JUP* | 2.079E-09 | *CD81* | 4.035E-18 |
| *P4HB* | 3.351E-15 | *HSPA8* | 2.029E-13 | *TUBA1A* | 4.360E-29 |
| *CEACAM6* | 6.077E-13 | *FTL* | 1.619E-20 | *FLNA* | 1.836E-10 |
| *FTL* | 1.597E-20 | *ALDOA* | 3.376E-15 | *CLU* | 3.944E-17 |
| *ALDOA* | 3.216E-15 | *S100A9* | 9.933E-13 | *IGFBP7* | 2.989E-16 |
| *C3* | 2.176E-13 | *NDRG1* | 9.728E-13 | *GPM6B* | 4.439E-08 |
| *CD63* | 9.534E-18 | *FLNA* | 1.343E-07 | *SPARC* | 1.298E-11 |
| *FLNA* | 1.325E-07 | *S100A11* | 1.839E-13 | *NES* | 1.480E-13 |
| *SPARC* | 4.750E-10 | *KRT17* | 2.327E-12 | *APP* | 1.595E-06 |
| *HSP90AB1* | 1.694E-09 | *SPARC* | 5.029E-10 | *CD63* | 9.474E-19 |
| *APLP2* | 2.557E-09 | *HSP90AB1* | 1.792E-09 | *ALDOA* | 1.208E-12 |
| *CANX* | 1.740E-15 | *PGK1* | 1.622E-10 | *ACTB* | 1.091E-13 |
| *VIM* | 1.001E-12 | *KRT5* | 1.323E-07 | *COL1A2* | 2.962E-14 |
| *GLUL* | 2.978E-09 | *GLUL* | 3.145E-09 | *CALR* | 2.163E-15 |
| *CALR* | 8.774E-13 | *SLC2A1* | 6.172E-08 | *VIM* | 1.798E-13 |
| *CTSB* | 1.862E-14 | *CALR* | 9.856E-13 | *CHI3L1* | 2.054E-15 |
| *PABPC1* | 3.860E-12 | *CTSB* | 2.202E-14 | *CD74* | 1.243E-12 |
| *KRT18* | 2.481E-10 | *PABPC1* | 4.104E-12 | *CANX* | 1.277E-11 |
| *ACTG1* | 6.504E-13 | *KRT14* | 5.620E-13 | *CTSB* | 1.083E-15 |
| *PIGR* | 1.136E-14 | *ACTG1* | 7.326E-13 | *MYL6* | 1.340E-07 |
| *COL3A1* | 3.334E-10 | *COL3A1* | 3.533E-10 | *MME* | 5.566E-11 |
| *COL1A2* | 1.238E-08 | *COL1A2* | 1.306E-08 | *GLUL* | 2.343E-10 |
| *LGALS3BP* | 1.596E-08 | *HSPB1* | 5.633E-08 | *MT3* | 1.176E-13 |
| *LYZ* | 4.403E-08 | *CD9* | 3.188E-10 | *ACTG1* | 4.093E-14 |
| *SFTPA2* | 6.670E-07 | *SFTPA2* | 6.761E-07 | *FTH1* | 3.312E-09 |
| *SFTPA1* | 2.748E-07 | *NAT1* | 7.000E-05 | *HLA-C* | 3.859E-10 |
| *NAT1* | 6.686E-05 | *B2M* | 2.868E-05 | *PLP1* | 6.797E-11 |
| *B2M* | 2.738E-05 | *FTH1* | 2.663E-05 | *QKI* | 2.088E-08 |
| *FTH1* | 2.541E-05 | *FN1* | 8.189E-04 | *PCDHGC3* | 8.640E-07 |
| *FN1* | 7.717E-04 | *CTSD* | 2.598E-04 | *SERPINA3* | 2.780E-02 |
| *CTSD* | 2.446E-04 | *KRT15* | 2.935E-02 | *BRI3* | 2.782E-08 |
| *LPCAT1* | 2.506E-06 | *SDC1* | 2.798E-08 | *HSPA8* | 1.357E-09 |
| *SLC34A2* | 7.538E-05 | *LDHA* | 2.833E-07 | *AQP4* | 3.333E-07 |
| *SERPINA1* | 3.826E-06 | *GSTP1* | 1.246E-02 | *GFAP* | 1.795E-06 |
| *LDHA* | 2.794E-07 | *ANXA2* | 8.001E-05 | *NAT1* | 1.356E-07 |
| *ANXA2* | 7.646E-05 | *MYH9* | 4.997E-02 | *B2M* | 6.925E-04 |
| *MYH9* | 4.997E-02 | *HLA-C* | 9.233E-06 | *IGFBP5* | 1.804E-14 |
| *HLA-C* | 8.805E-06 | *AKR1C1* | 1.819E-02 | *CRYAB* | 9.417E-04 |
| *YWHAZ* | 4.506E-02 | *YWHAZ* | 4.502E-02 | *LRP1* | 1.732E-08 |
| *HLA-A* | 6.246E-03 | *KRT13* | 1.886E-03 | *HLA-A* | 3.333E-07 |
| *COL1A1* | 1.304E-01 | *HLA-A* | 6.523E-03 | *SPARCL1* | 1.083E-04 |
| *HLA-DRA* | 6.860E-01 | *COL1A1* | 1.303E-01 | *S100B* | 1.872E-04 |
|  |  | *HLA-DRA* | 6.859E-01 | *FN1* | 4.728E-02 |
|  |  |  |  | *CST3* | 6.713E-04 |
|  |  |  |  | *PMP2* | 4.811E-02 |
|  |  |  |  | *ITM2B* | 1.148E-01 |
|  |  |  |  | *HLA-DRA* | 6.206E-01 |

**Table S7. Genes in LUAD-PGS.**

| **Gene Symbol** | **Full Gene Name** |
| --- | --- |
| *ACTB* | Actin Beta |
| *FTL* | Ferritin Light Chain |
| *SFTPA2* | Surfactant Protein A2 |
| *CD74* | Cluster of Differentiation 74 Molecule |
| *FN1* | Fibronectin 1 |
| *B2M* | Beta-2-Microglobulin |
| *CTSD* | Cathepsin D |
| *CEACAM6* | Carcinoembryonic Antigen-Related Cell Adhesion Molecule 6 |
| *EEF2* | Eukaryotic Translation Elongation Factor 2 |
| *PGC* | Progastricsin |
| *UBC* | Ubiquitin C |
| *HSP90AB1* | Heat Shock Protein 90 Alpha Family Class B Member 1 |
| *SERPINA1* | Serpin Family A Member 1 |
| *HSPA8* | Heat Shock Protein Family A (Hsp70) Member 8 |
| *HSP90AA1* | Heat Shock Protein 90 Alpha Family Class A Member 1 |
| *GNB2L1* (*RACK1*) | Receptor For Activated C Kinase 1 |
| *CEACAM5* | Carcinoembryonic Antigen-Related Cell Adhesion Molecule 5 |
| *CD63* | Cluster of Differentiation 63 Molecule |
| *PIGR* | Polymeric Immunoglobulin Receptor |
| *KRT18* | Keratin 18 |
| *GLUL* | Glutamate-Ammonia Ligase |
| *KRT19* | Keratin 19 |

**Table S8. Genes in LUSC-PGS.**

| **Gene Symbol** | **Full Gene Name** |
| --- | --- |
| *GAPDH* | Glyceraldehyde-3-Phosphate Dehydrogenase |
| *KRT5* | Keratin 5 |
| *ACTG1* | Actin Gamma 1 |
| *ENO1* | Enolase 1 |
| *PKM* | Pyruvate Kinase M1/2 |
| *CTSB* | Cathepsin B |
| *PSAP* | Prosaposin |
| *MYH9* | Myosin Heavy Chain 9 |
| *KRT14* | Keratin 14 |
| *RPS4X* | Ribosomal Protein S4 X-Linked |
| *CALR* | Calreticulin |
| *FLNA* | Filamin A |
| *HSPA8* | Heat Shock Protein Family A (Hsp70) Member 8 |
| *SFTPA2* | Surfactant Protein A2 |
| *RPS11* | Ribosomal Protein S11 |
| *HSP90B1* | Heat Shock Protein 90 Beta Family Member 1 |
| *HSPB1* | Heat Shock Protein Family B (Small) Member 1 |
| *SDC1* | Syndecan 1 |
| *HLA-C* | Major Histocompatibility Complex, Class I, C |
| *APP* | Amyloid Beta Precursor Protein |
| *ATP1A1* | ATPase Na+/K+ Transporting Subunit Alpha 1 |
| *HSPA5* | Heat Shock Protein Family A (Hsp70) Member 5 |
| *RPL37* | Ribosomal Protein L37 |

**Table S9. Genes in GBM-PGS.**

| **Gene Symbol** | **Full Gene Name** |
| --- | --- |
| *RPS11* | Ribosomal Protein S11 |
| *UBB* | Ubiquitin B |
| *TUBB* | Tubulin Beta Class I |
| *RPS6* | Ribosomal Protein S6 |
| *EEF1A1* | Eukaryotic Translation Elongation Factor 1 Alpha 1 |
| *EEF2* | Eukaryotic Translation Elongation Factor 2 |
| *PKM* | Pyruvate Kinase M1/2 |
| *C3* | Complement C3 |
| *ENO1* | Enolase 1 |
| *HSP90AB1* | Heat Shock Protein 90 Alpha Family Class B Member 1 |
| *FTL* | Ferritin Light Chain |
| *CFL1* | Cofilin 1 |
| *YWHAE* | Tyrosine 3-Monooxygenase/Tryptophan 5-Monooxygenase Activation Protein Epsilon |
| *CKB* | Creatine Kinase B |
| *TUBA1A* | Tubulin Alpha 1A |
| *FLNA* | Filamin A |
| *APP* | Amyloid Beta Precursor Protein |
| *CD63* | Cluster of Differentiation 63 Molecule |
| *ACTB* | Actin Beta |
| *VIM* | Vimentin |
| *CTSB* | Cathepsin B |
| *MME* | Membrane Metalloendopeptidase |
| *GLUL* | Glutamate-Ammonia Ligase |
| *MT3* | Metallothionein 3 |
| *ACTG1* | Actin Gamma 1 |
| *HLA-C* | Major Histocompatibility Complex, Class I, C |
| *B2M* | Beta-2-Microglobulin |
| *CRYAB* | Crystallin Alpha B |
| *LRP1* | Low-Density Lipoprotein Receptor Related Protein 1 |
| *S100B* | S100 Calcium Binding Protein B |
| *FN1* | Fibronectin 1 |

**Table S10. Frequency and prognostic significance of mutations in LUAD-PGS genes in the TCGA LUAD cohort.** Kaplan-Meier survival curves analyzed disease-free survival times between mutant or wild-type patients for each PGS gene. *P*-values calculated using log-rank tests are shown. Genes with mutations significantly correlated with patient prognosis are highlighted in grey.

| **Gene** | **Mutant Patients** | **Wild-Type Patients** | **KM log-rank *P*-value** |
| --- | --- | --- | --- |
| *ACTB* | 12 | 173 | 0.2126 |
| *FTL* | 2 | 183 | 0.5284 |
| *SFTPA2* | 1 | 184 | 0.4086 |
| *CD74* | 1 | 184 | 0.8161 |
| *FN1* | 7 | 178 | 0.3636 |
| *B2M* | 6 | 179 | 0.3174 |
| *CTSD* | 0 | 185 | N/A |
| *CEACAM6* | 3 | 182 | 0.3841 |
| ***EEF2*** | **3** | **182** | **0.0022** |
| *PGC* | 12 | 173 | 0.6639 |
| *UBC* | 8 | 177 | 0.1792 |
| *HSP90AB1* | 12 | 173 | 0.2348 |
| *SERPINA1* | 3 | 182 | 0.1377 |
| *HSPA8* | 7 | 178 | 0.8296 |
| *HSP90AA1* | 7 | 178 | 0.1986 |
| *GNB2L1* (*RACK1*) | 1 | 184 | 0.3223 |
| *CEACAM5* | 6 | 179 | 0.1066 |
| *CD63* | 4 | 181 | 0.7312 |
| *PIGR* | 15 | 170 | 0.8551 |
| *KRT18* | 3 | 182 | 0.9135 |
| *GLUL* | 15 | 170 | 0.7759 |
| *KRT19* | 1 | 184 | 0.2955 |

**Table S11. Frequency and prognostic significance of mutations in LUSC-PGS genes in the TCGA LUSC cohort.** Kaplan-Meier survival curves analyzed disease-free survival times between mutant or wild-type patients for each PGS gene. *P*-values calculated using log-rank tests are shown. Genes with mutations significantly correlated with patient prognosis are highlighted in grey.

| **Gene** | **Mutant Patients** | **Wild-Type Patients** | **KM log-rank *P*-value** |
| --- | --- | --- | --- |
| *GAPDH* | 9 | 112 | 0.6864 |
| *KRT5* | 0 | 121 | N/A |
| *ACTG1* | 8 | 113 | 0.2008 |
| *ENO1* | 1 | 120 | 0.8653 |
| *PKM* | 5 | 116 | 0.1453 |
| ***CTSB*** | **8** | **113** | **< 0.0001** |
| *PSAP* | 4 | 117 | 0.1688 |
| *MYH9* | 8 | 113 | 0.5038 |
| *KRT14* | 6 | 115 | 0.4735 |
| *RPS4X* | 2 | 119 | 0.5029 |
| *CALR* | 2 | 119 | 0.3733 |
| *FLNA* | 12 | 109 | 0.0689 |
| *HSPA8* | 3 | 118 | 0.5524 |
| *SFTPA2* | 8 | 113 | 0.5463 |
| *RPS11* | 3 | 118 | 0.9702 |
| ***HSP90B1*** | **2** | **119** | **< 0.0001** |
| *HSPB1* | 1 | 120 | 0.6272 |
| *SDC1* | 4 | 117 | 0.7084 |
| *HLA-C* | 1 | 120 | N/A |
| *APP* | 5 | 116 | 0.9992 |
| *ATP1A1* | 5 | 116 | 0.2789 |
| *HSPA5* | 2 | 119 | 0.3398 |
| *RPL37* | 21 | 100 | 0.1694 |

**Table S12. Frequency and prognostic significance of mutations in GBM-PGS genes in the TCGA GBM cohort.** Kaplan-Meier survival curves analyzed disease-free survival times between mutant or wild-type patients for each PGS gene. *P*-values calculated using log-rank tests are shown. Genes with mutations significantly correlated with patient prognosis are highlighted in grey.

| **Gene** | **Mutant Patients** | **Wild-Type Patients** | **KM log-rank *P*-value** |
| --- | --- | --- | --- |
| *RPS11* | 0 | 191 | N/A |
| *UBB* | 0 | 191 | N/A |
| *TUBB* | 0 | 191 | N/A |
| *RPS6* | 2 | 189 | 0.7997 |
| *EEF1A1* | 2 | 189 | 0.6429 |
| *EEF2* | 2 | 189 | 0.2193 |
| *PKM* | 1 | 190 | 0.1026 |
| *C3* | 4 | 187 | 0.9628 |
| *ENO1* | 2 | 189 | 0.2802 |
| *HSP90AB1* | 2 | 189 | 0.6302 |
| *FTL* | 0 | 191 | N/A |
| *CFL1* | 1 | 190 | 0.5808 |
| *YWHAE* | 1 | 190 | 0.1983 |
| *CKB* | 1 | 190 | 0.5163 |
| *TUBA1A* | 2 | 189 | 0.1250 |
| *FLNA* | 8 | 183 | 0.6733 |
| ***APP*** | **1** | **190** | **0.0033** |
| *CD63* | 1 | 190 | 0.5421 |
| *ACTB* | 3 | 188 | 0.1451 |
| *VIM* | 0 | 191 | N/A |
| *CTSB* | 1 | 190 | 0.1589 |
| ***MME*** | **1** | **190** | **0.0198** |
| *GLUL* | 1 | 190 | 0.6717 |
| *MT3* | 0 | 191 | N/A |
| *ACTG1* | 0 | 191 | N/A |
| *HLA-C* | 0 | 191 | N/A |
| *B2M* | 0 | 191 | N/A |
| *CRYAB* | 2 | 189 | 0.9699 |
| *LRP1* | 9 | 182 | 0.7616 |
| *S100B* | 0 | 191 | N/A |
| *FN1* | 2 | 189 | 0.4096 |

**SUPPLEMENTARY FIGURES**


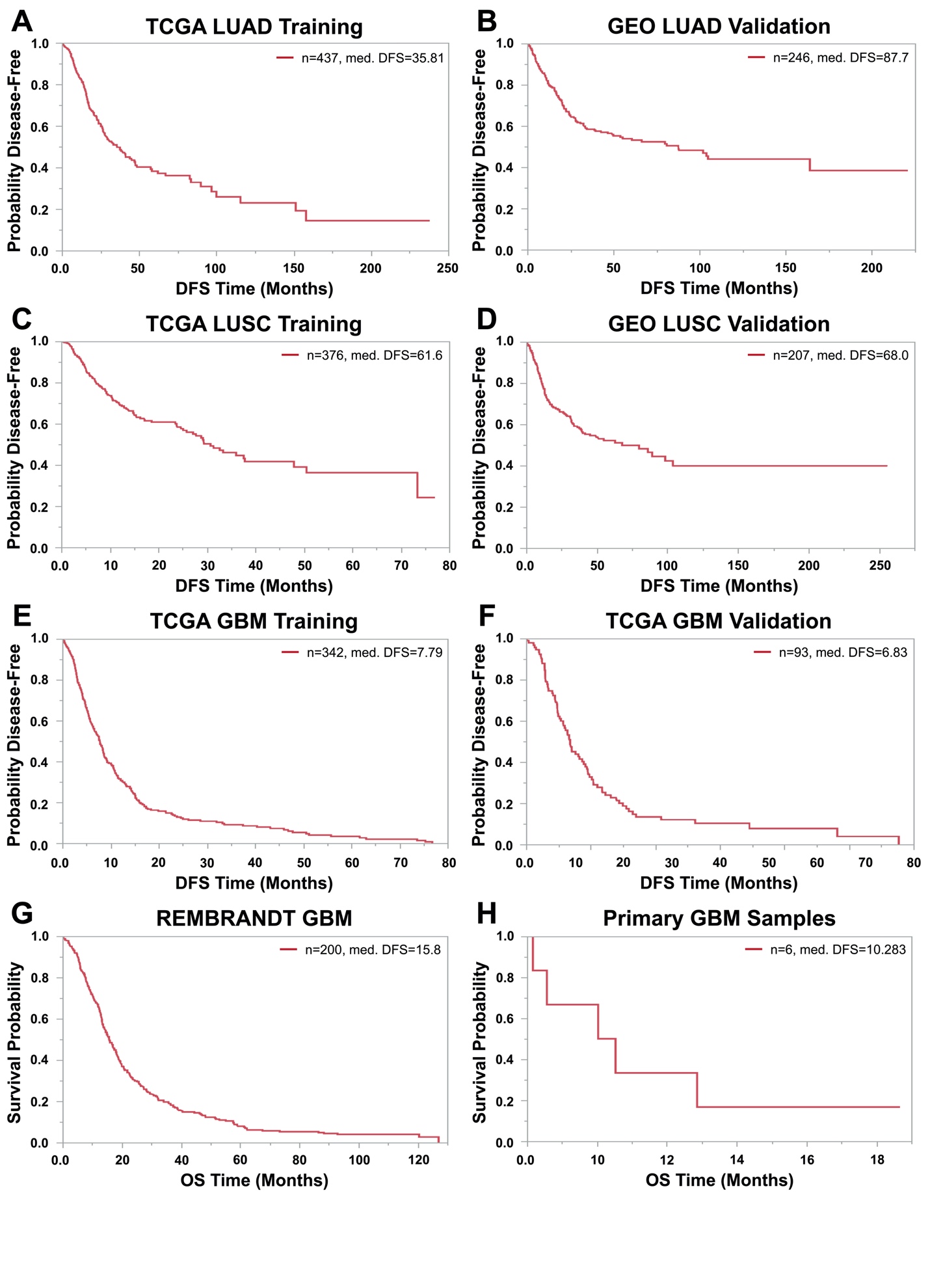


**Figure S1. Unstratified survival of training and validation cohorts.** Kaplan-Meier survival curves of all patients in training and validation cohorts for LUAD **[A,B]**, LUSC **[C,D]**, and GBM **[E-H]** are shown. Median DFS or OS times are shown in months. DFS—disease-free survival, OS—overall survival.


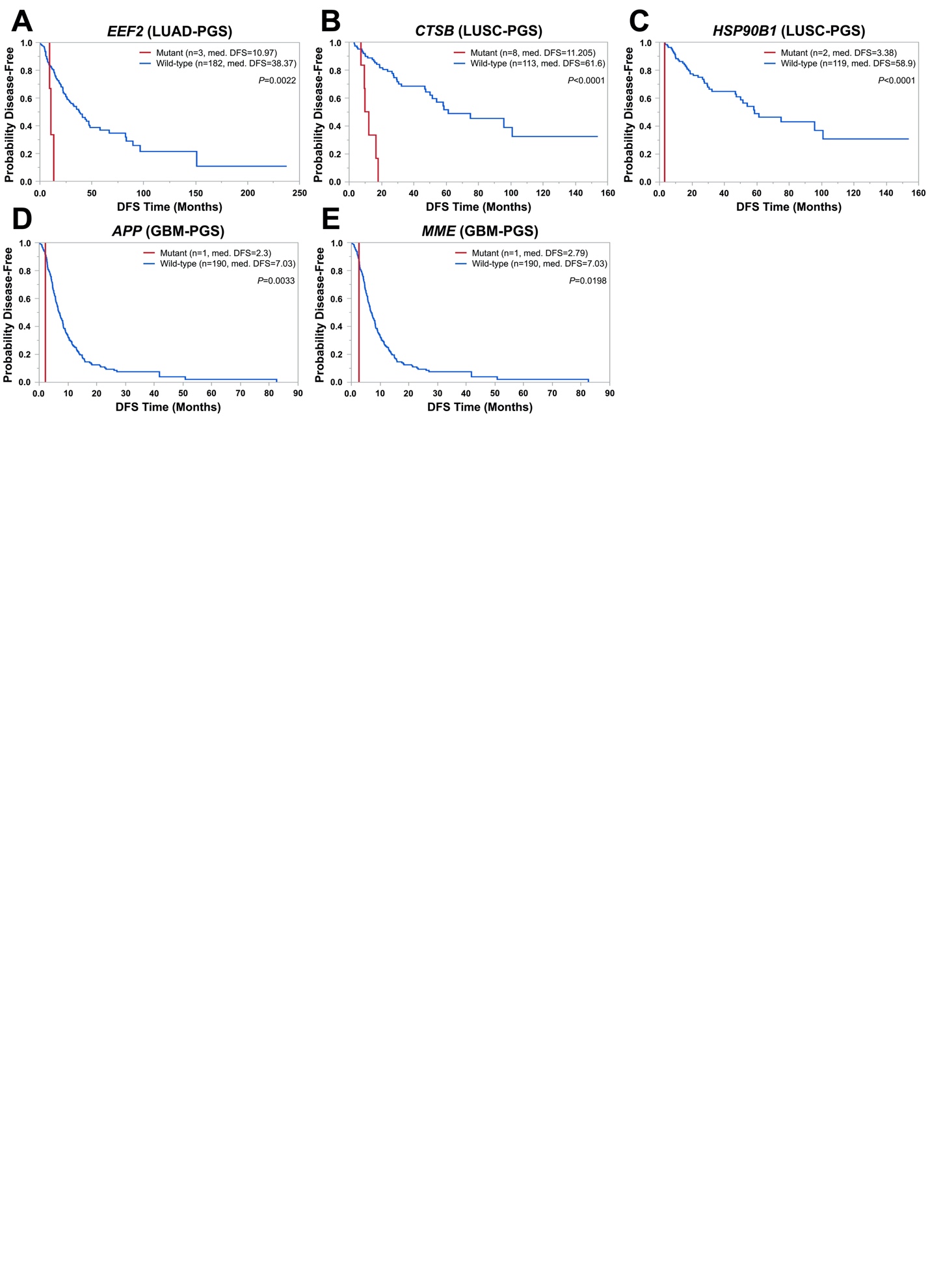


**Figure S2. Mutations in PGS genes correlate with poor patient prognosis.** Kaplan-Meier survival curves of disease-free survival (DFS) time in patients with mutant or wild-type *EEF2* in LUAD **[A]**, *CTSB* **[B]** or *HSP90B1* **[C]** in LUSC, and *APP* **[D]** or *MME* **[E]** in GBM. Median DFS times are shown in months. *P*-values were calculated using log-rank tests.


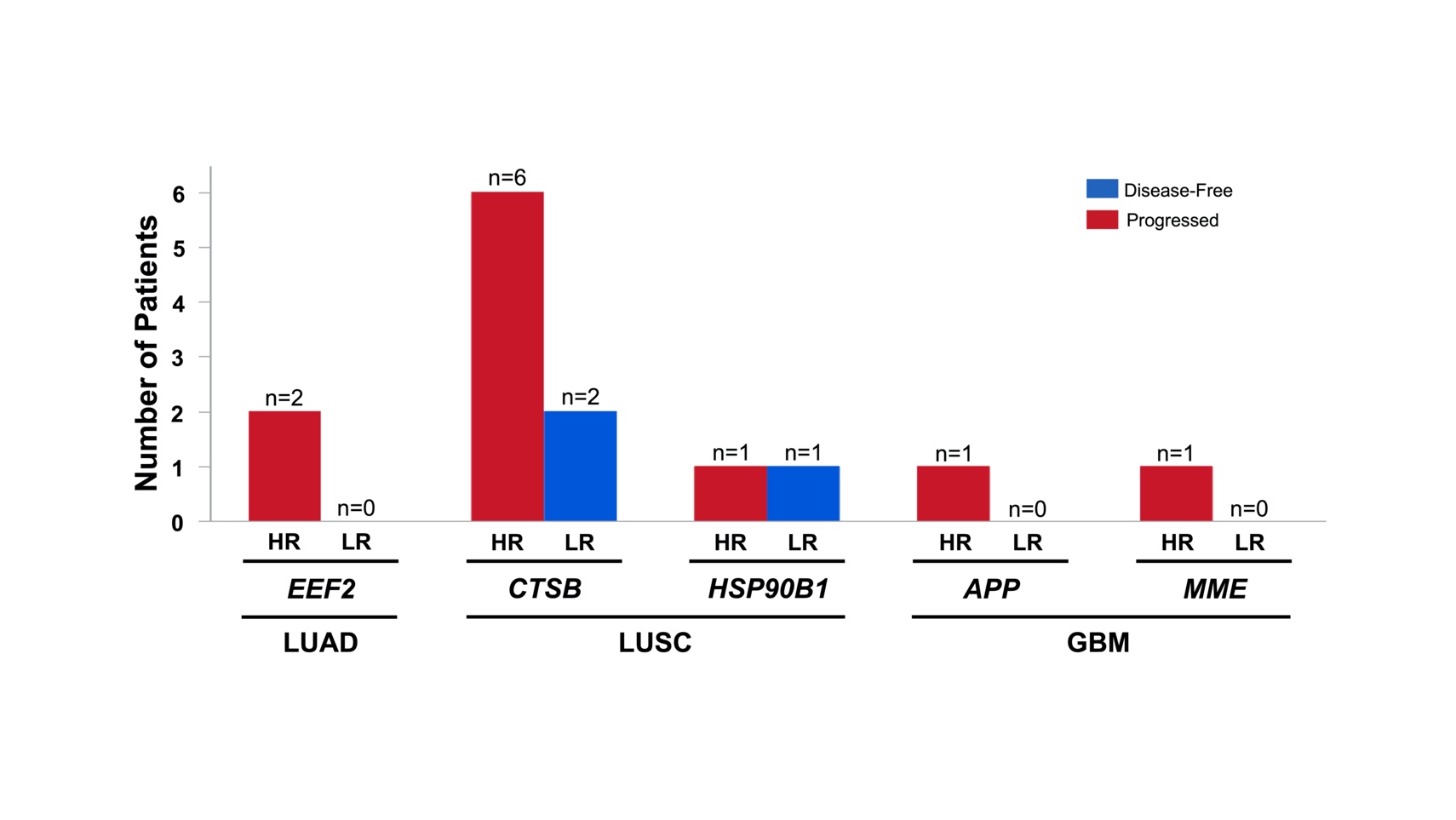


**Figure S3. Correlation of mutations in PGS genes with PGS risk groups.** Frequencies of high-risk progression (HR) or low-risk progression (LR) stratification in patients with mutant PGS genes are shown. The DFS status of patients in each risk group are shown as blue (disease-free) or red (progressed).


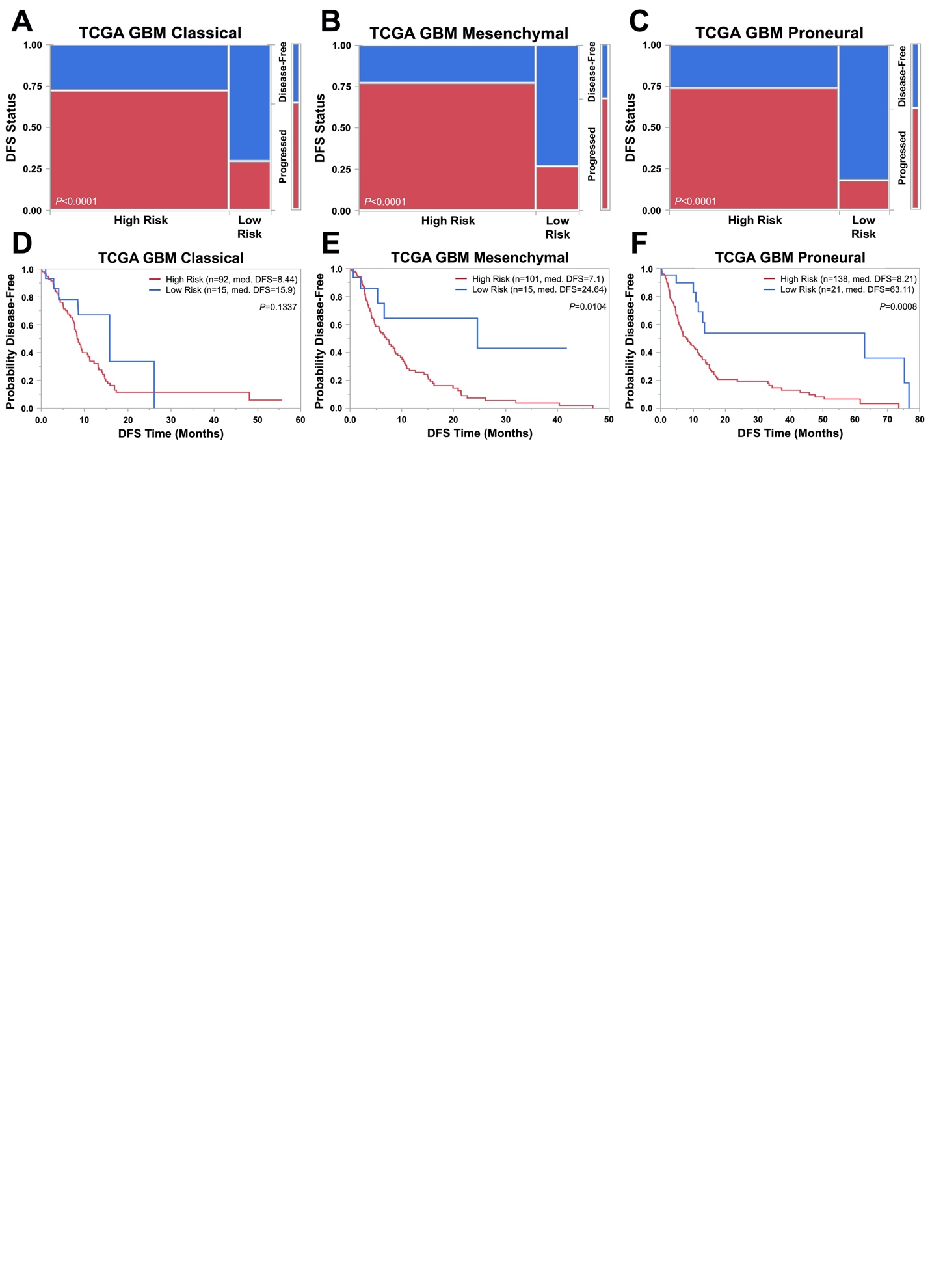


**Figure S4. PGS risk groups correlate with tumor progression incidence and disease-free survival in GBM subtypes.** **[A-C]** Patients stratified as high-risk progression (risk score > 0) or low-risk progression (risk score < 0) by GBM-PGS were analyzed for correlations with tumor progression incidence. Fisher’s Exact Tests determined significance of correlation. **[D-F]** Kaplan-Meier survival curves of disease-free survival (DFS) time between high- and low-risk patients. Median DFS times for each risk group are shown in months. *P*-values were calculated using log-rank tests. DFS—disease-free survival.


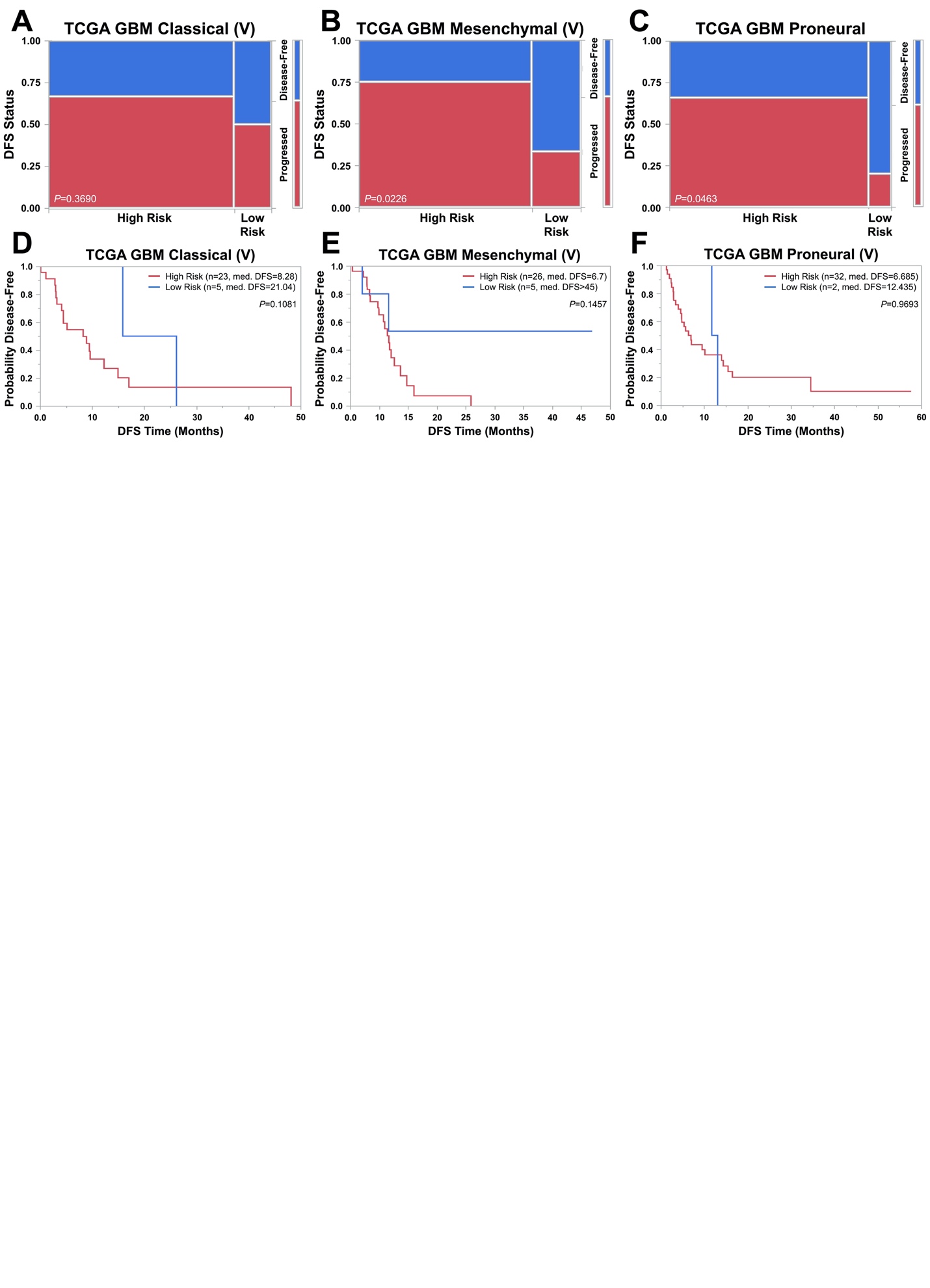


**Figure S5. Correlation of PGS risk groups with tumor progression in GBM subtypes in the TCGA GBM validation cohort.** **[A-C]** Patient risk stratification by GBM-PGS in each GBM subtype in the 126-patient TCGA GBM validation cohort. *P*-values were calculated via Fisher’s Exact Tests. **[D-F]** Kaplan-Meier survival curves of disease-free survival (DFS) time between high- and low-risk patients. Median DFS times for each risk group are shown in months. *P*-values were calculated using log-rank tests. DFS—disease-free survival.
